# Approach to nigericin derivatives and their therapeutic potential†

**DOI:** 10.1039/d0ra05137c

**Published:** 2020-11-26

**Authors:** Amit Kumar Sahu, Madhukar S. Said, Tejashri Hingamire, Megha Gaur, Abujunaid Khan, Dhanasekaran Shanmugam, Vitthal T. Barvkar, Mahesh S. Dharne, Atul A. Bharde, Syed G. Dastager

**Affiliations:** Academy of Scientific and Innovative Research (AcSIR), CSIR National Chemical Laboratory Pune-411008 India sg.dastager@ncl.res.in; National Collection of Industrial Microorganisms (NCIM), CSIR National Chemical Laboratory Pune-411008 India; Biochemical Sciences Division, CSIR National Chemical Laboratory Pune-411008 India; Organic Chemistry Division, CSIR National Chemical Laboratory Pune-411008 India; Department of Botany, Savitribai Phule-Pune University Pune-411007 India; Department of Microbiology, Savitribai Phule-Pune University Pune-411007 India

## Abstract

A new nigericin analogue that has been chemically modified was synthesized through a fluorination process from the parent nigericin, produced from a novel *Streptomyces* strain DASNCL-29. Fermentation strategies were designed for the optimised production of nigericin molecule and subjected for purification and structural analysis. The fermentation process resulted in the highest yield of nigericin (33% (w/w)). Initially, nigericin produced from the strain DASNCL-29 demonstrated polymorphism in its crystal structure, *i.e.*, monoclinic and orthorhombic crystal lattices when crystallised with methanol and hexane, respectively. Furthermore, nigericin produced has been subjected to chemical modification by fluorination to enhance its efficacy. Two fluorinated analogues revealed that they possess a very potent antibacterial activity against Gram positive and Gram negative bacteria. To date, the nigericin molecule has not been reported for any reaction against Gram-negative bacteria, which are increasingly becoming resistant to antibiotics. For the first time, fluorinated analogues of nigericin have shown promising activity. *In vitro* cytotoxicity analysis of fluorinated analogues demonstrated tenfold lesser toxicity than the parent nigericin. This is the first type of study where the fluorinated analogues of nigericin showed very encouraging activity against Gram-negative organisms; moreover, they can be used as a candidate for treating many serious infections.

## Abstract 2

A new nigericin analogue that has been chemically modified was synthesized through a fluorination process from the parent nigericin, produced from a novel *Streptomyces* strain DASNCL-29.

## Graphical abstract

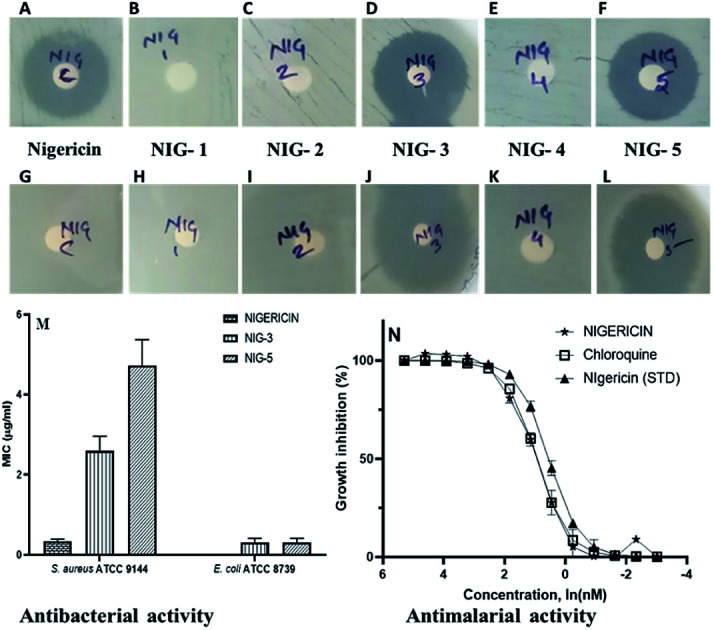

## Introduction

Antimicrobial resistance is increasing and received global concern as mortality due to infections caused by resistant pathogens is on an upsurge in all countries.^1^ In the European region, 33 000 deaths are annually reported due to AMR. Infants across the globe, mostly in developing countries, are affected by infections because of resistant pathogens.^2^ Unfortunately, we entered a post-antibiotic era where people are dying because of infections caused by resistant pathogens, especially the resistance among Gram-negative pathogens, which is a considerable threat.^3^ In 2019, the World Health Organisation published a list of high priority pathogens, which are a threat to human health. In that list, the majority are Gram-negative pathogens resistant to one or other antibiotics used to kill them. At present, we do not have any novel therapeutic candidate to fight against Gram-negative pathogens. By 2050, if the resistance pattern keeps growing at the same rate, the mortality due to resistance infections will have reached 10 million people per year, and this cause will be higher than any other cause of deaths such as road accidents and cancer.^4^ Nevertheless, we hope to tackle this condition by speeding up the discovery of novel molecules from various sources and combining different drug discovery strategies. Previously, chemical modification such as fluorination of numerous drug candidates has been exploited extensively in drug design and development because molecules containing fluorine possibly influence conformation, p*K*_a_, intrinsic potency, membrane permeability, metabolic pathways, and pharmacokinetic properties.^5^

Nigericin is a polyether ionophore; it is reported to have a very potent antibacterial, antifungal, antiviral, antimalarial and anticancer activity.^6–9^ Nigericin is produced mainly by the *Streptomyces* species. It was first isolated from *Streptomyces hygroscopicus* in the 1950s, and its complex structure was revealed using X-ray crystallography.^10^ It possesses a high affinity for monovalent cations such as Na^+^ and K^+^ and is also known to disrupt the ionic balance, thereby affecting the transmembrane potential across the plasma membrane by selectively transporting cations.^11^ It was also found to inhibit glucose transport in adipocytes.^12^ Various studies have attributed nigericin with a broad spectrum of biological activities, including antibacterial activity against Gram-positive microorganisms and antifungal activity. It also enhances the antifungal activity of rapamycin.^13^ Nigericin is reported to be a potent anti-cancer compound because it selectively inhibits the growth of many cancer cells.^14,15^ Nigericin possesses very promising antimalarial potential because it kills the malarial parasite at a very low effective concentration^16–18^ by disrupting the ion homeostasis.^19^ It is a potent inhibitor of viruses such as vaccinia,^20^ human immuno-deficiency virus (HIV)^21^ and polio virus;^22^ moreover, nigericin has been demonstrated to enhance the internalisation of drugs.^23,24^

A total of 53 strains of the Streptomycetaceae family have been reported to produce different ionophores. These microorganisms belong to three types of genera, *i.e.*, the *Streptomyces*, *Actinomodura* and *Dactylosporangium*. In fact, >50% of ionophores identified to date were derived from two *Streptomyces* species (*Streptomyces hygroscopicus* and *Streptomyces albus*). Industrially, biologically active molecules can be produced by microorganisms at a low cost.^25^ Nigericin was among the first polyether ionophores to be discovered; however, its chemical synthesis remains obscure due to the low yield of the molecule from *Streptomyces hygroscopicus* BRM10 and was obtained as a by-product/contaminant during the fermentation of geldanamycin.^8,26–28^

In this study, a novel indigenous species of *Streptomyces* strain DASNCL-29 (Fig. S1–S4 and Tables S1–S4†) was isolated from a plant-associated soil sample, collected from Unkeshwar, Maharashtra, India, and it was found to produce nigericin. For the first time, it was observed that it generated nigericin as the major secondary metabolite, which is not the case with earlier reports of nigericin. We could also assess the yield at a pilot scale. The purified polymorphic crystalline form of the molecule was subjected to an array of known bioactivity studies. Later, the molecule was improved by chemical modification, *i.e.*, fluorination and evaluating the efficacy of derivatives compared with the parent molecule.

## Results and discussion

### Fermentation and structure elucidation of bioactive compounds

Submerged fermentation was performed for the strain DASNCL-29 in a semi-automatic BioFlo 115 bioreactor. Fermentation conditions are provided in Table S5B.† This fermentation yielded a 25.0 g of a pure white crystalline molecule from 75.0 g of crude extract that was achieved by a 50 L fermenter, which accounts for 33% (w/w) of the yield. The optimised conditions for the strain DASNCL-29 afforded a yield of 500 mg L^−1^ of pure compound. In previous studies, nigericin was isolated as a by-product during the fermentation of geldanamycin. *Streptomyces* sp. DASNCL-29 produces nigericin, which is 2 to 10 fold higher yield than the previous reports of nigericin yield of 50–100 μg ml^−1^, 0.1 mg ml^−1^, and 0.318 mg ml^−1^ from the *Streptomyces* species, namely *S. albus* and *S. hygroscopicus*,^26,28,29^ respectively. In a recent study, a *Streptomyces* co-culturing methodology was shown to produce a comparable amount of nigericin (0.49 mg ml^−1^) using solid-state fermentation (SSF). However, the reported strain SF10 was unable to produce nigericin under submerged fermentation conditions.^30^ To develop scaling up strategies for nigericin at a commercially viable scale, submerged fermentation is more conducive as it provides scope for further improvement. In contrast, the SSF raised several engineering problems due to the fermentation design and parameter control, such as dissolved oxygen, pH and temperature, and complicated the down streaming process further.^31,32^ The purified molecule appeared as a white crystalline powder subjected for analysis of HMBC, NOSY and COSY spectral correlations, as shown in Fig. S5.† Detailed Information on 1D and 2D NMR of the bioactive compound is available in the ESI (S15 and S16)† and the molecular formula of C_40_H_68_O_11_. In HR-ESI-MS analysis, a peak was detected at 724.96 [M + 1]^+^, indicating seven sites of unsaturation. An earlier report of the crystallographic structure of nigericin had shown an orthorhombic crystal system.^10^ When the molecular structure of the bioactive compound was determined by single X-ray crystallography, a polymorphic structure of nigericin was observed, which was not previously reported, where crystallisation in methanol formed a monoclinic (Fig. S6A and CCDC 1946291†) space group crystal system, space group *P*2_1_ with *a* = 14.46(8) Å, *b* = 8.49(5) Å and *c* = 16.64(10) Å, whereas *α* = 90°, *β* = 103.03(2)° and *γ* = 90°. However, on recrystallization from hexane, nigericin formed an orthorhombic (Fig. S6, CCDC 1946293†) space group crystal system, space group *P*2_1_2_1_2_1_ with *a* = 12.02(3) Å, *b* = 14.46 Å and *c* = 20.09(8) Å with *α*, *β* and *γ* = 90°. The mass spectrum for nigericin from strain DASNCL-29, together with standard nigericin (Sigma, USA), is shown in Fig. S17–S19.†

### Synthesis of fluorinated nigericin analogues

**Scheme 1:**
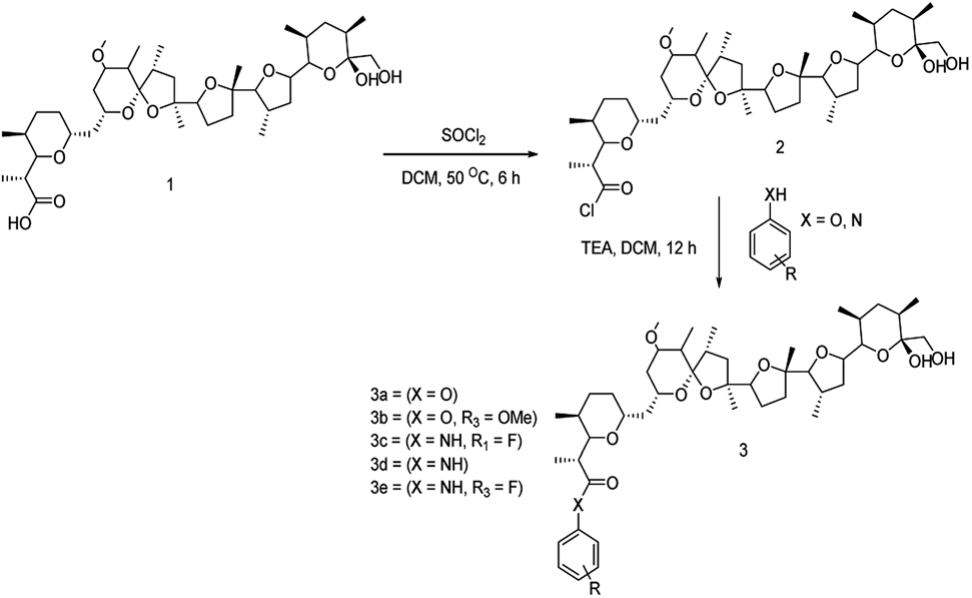
Reaction scheme for synthesis of nigericin derivatives.

The structure of nigericin was chemically tailored and modified to improve its structural, biological and pharmacological properties. The chemical synthesis strategies adopted, which involved fluorination and esterification, as shown in Scheme 1, resulted in five different analogues. However, out of the five, two of the analogues were fluorine-containing, and the remaining three were having an additional benzene ring attached to the carboxyl group (Fig. S8 and S9†). The chemically synthesized analogues were further characterised for their structure and presence of fluorine moiety using NMR spectroscopy and HRMS data as mentioned below (Table S6†).

### Bioactivity studies on nigericin and its derivatives

**Fig. 1:**
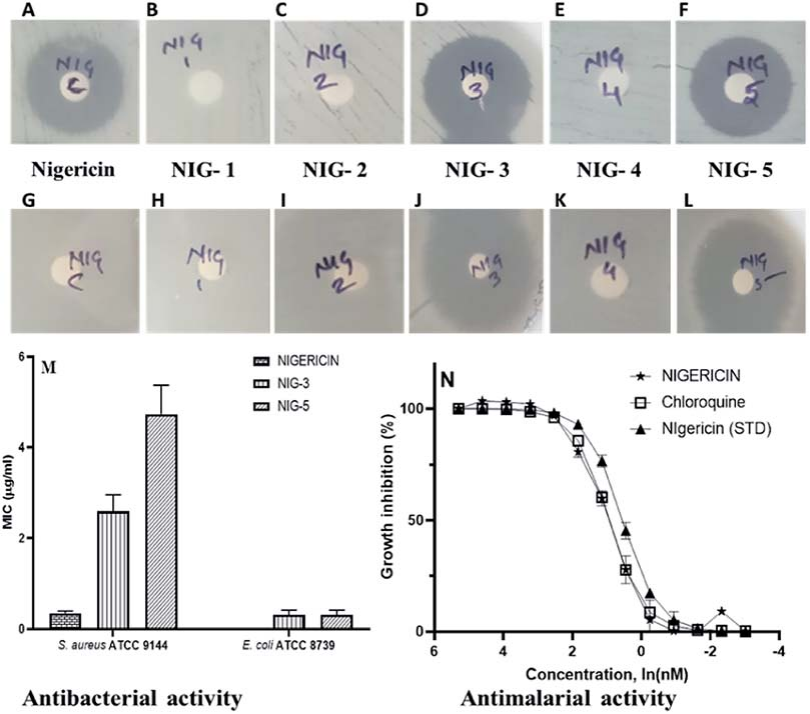
Antibacterial activity (disc diffusion method). (A–F) Antibacterial against Gram-positive test strain (*S. aureus* ATCC 9144), (G–L) antibacterial against Gram-negative test strain (*E. coli* ATCC 8739). (M) Minimum inhibitory concentration of nigericin and its fluorinated analogs. Nigericin is represented as Nig-C, fluorinated analog 3 is represented as Nig-3, and fluorinated analog 5 is represented as Nig-5. (N) Determining the EC_50_ values for inhibition of blood-stage malaria parasite growth. The number of replicates *n* = 3 for all samples. Standard nigericin was procured from Sigma Aldrich.

Nigericin has been reported to have an inhibitory effect on prokaryotic and eukaryotic organisms.^33,34^ We have analysed the antibacterial and antimalarial activity of the nigericin isolated from an indigenous strain DASNCL-29 and its modified analogues for evaluating its potential application and commercial synthesis. As previously reported, it was observed that the parent molecule, *i.e.*, nigericin was only able to inhibit Gram-positive bacteria, as shown in Fig. S10.† However, it does not show antibacterial activity against any Gram-negative pathogens. In this study, the nigericin-derived fluorinated analogues, exclusively those containing a fluorine atom, were reported to inhibit the Gram positive and the Gram negative organisms (Fig. 1). The zones of inhibitions are tabulated in Table 1. In the present situation of antimicrobial resistance, there is an urgent requirement to tackle the antimicrobial resistance amongst Gram negative pathogens. Fluorinated analogues could be an excellent therapeutic candidate for use in the medicament of infection caused by these pathogens. High efficacy and low toxicity of derivative from a parent molecule attributes to a good therapeutic index of nigericin fluorinated analogues. Minimum inhibitory concentration (MIC) of nigericin was determined for Gram-positive test strain *S. aureus* ATCC 9144 (Fig. S10†), which has a MIC of 0.3 μM and no activity was observed towards Gram-negative test organism *E. coli* ATCC 8739 at the highest tested concentration. For fluorinated analogues, NIG-3 inhibited the growth of Gram-positive and Gram-negative test organisms with a MIC of 2.5 μM and 0.3 μM, respectively. NIG-5 also inhibited the growth of Gram-positive and Gram-negative test strains with a MIC of 5.0 μM and 0.3 μM, respectively. Transmission electron microscopy (TEM) observation clearly showed the intact ionophoric nature of the molecules because cell bursting was observed in the Gram-positive strain (*S. aureus* ATCC 9144) on treatment with nigericin and fluorinated analogues (NIG-3 and NIG-5). For Gram-negative strain (*E. coli* ATCC 8739), fluorinated analogues (NIG-3 and NIG-5) showed bursting of cells, which is similar to the action of parent molecule towards Gram-positive strains (Fig. S11†).

**Table 1:** Antibacterial activity of nigericin and its fluorinated analogues (zone measured in mm). Nigericin is represented as C whereas the fluorinated nigericin analogues are present as 1 to 5. (—, indicates no antibacterial activity observed)

Test organisms	Zone of inhibition of test compounds (in mm)					
	C	1	2	3	4	5
**Gram +ve organisms**						
*S. aureus* ATCC 9144	10.0	—	—	10.0	—	10.0
*S. epidermidis* ATCC 12228	15.0	—	—	11.0	—	11.0
*B. subtilis* ATCC 6051	10.0	—	—	12.0	—	11.0
*B. cereus* ATCC 10876	12.0	—	—	14.0	—	12.0

**Gram −ve organisms**						
*E. coli* ATCC 8739	—	—	—	20.0	—	12.0
*P. aeruginosa* ATCC 19154	—	—	—	11.0	—	11.0
*K. pneumoniae* ATCC 700603	—	—	—	—	—	—
*S. typhimurium* ATCC 23564	—	—	—	23.0	—	16.0

However, note that nigericin did not show any activity towards Gram-negative strain, wherein, its fluorinated analogs showed very potent activity. For the first time, nigericin analogues have shown activity against Gram-negative bacteria, which is very promising to address the drug resistance problem. Nigericin has previously been reported to have potent antimalarial activity with IC_50_ of 1.0 ng ml^−1^ and ∼100- to 200-fold less activity against two mammalian cell lines (50% viability of U937 macrophages at 193.0 ng ml^−1^ and 50% viability of Jurkat lymphoblast at 90.0 ng ml^−1^).^35^ Our interest here was to establish the antimalarial potency of nigericin isolated from strain DASNCL-29 and its analogues.

For antimalarial activity, we treated blood-stage *Plasmodium falciparum* (3D7 strain) with various concentrations ranging from 10 μM to 0.01 μM of nigericin and its analogues to estimate its dose-dependent inhibitory activity (further details of the antimalarial activity assays are given in the ESI†). The antimalarial EC_50_ value for the nigericin molecule was 2.4 nM (Fig. 1 and Table S7†). However, none of the nigericin analogues showed any inhibition towards blood-stage *Plasmodium falciparum* (3D7 strain) even at the highest tested concentration (10.0 μM).

### *In vitro* cytotoxicity of nigericin and its analogues

**Fig. 2:**
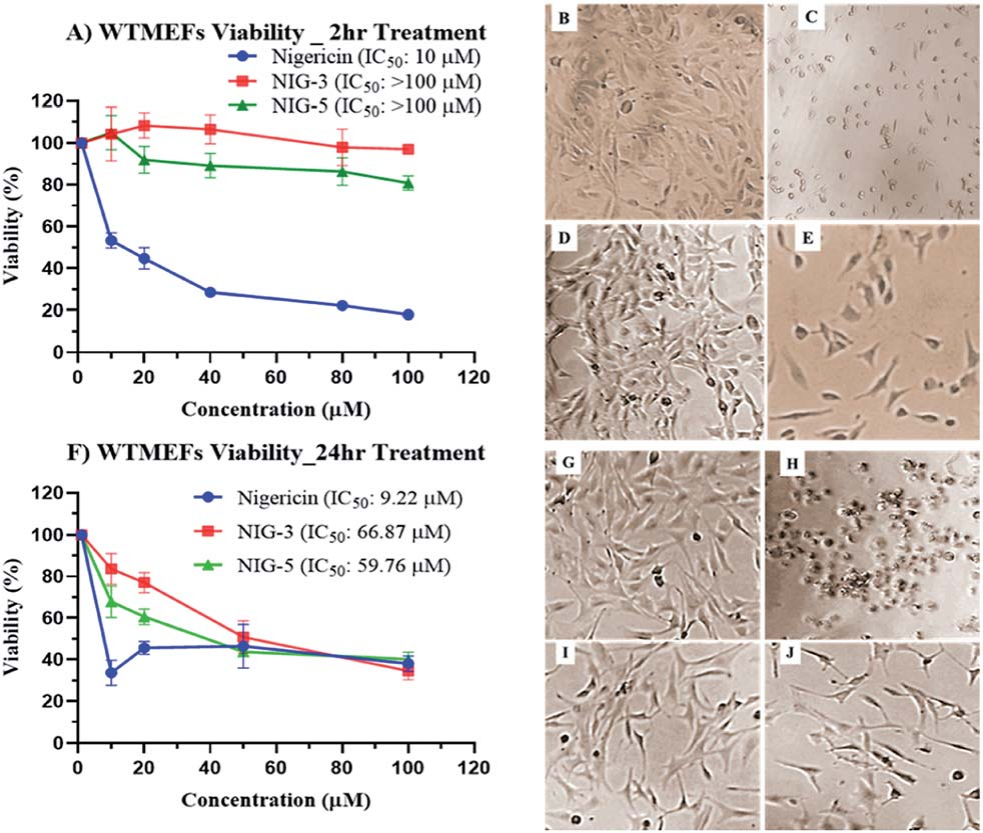
Cytotoxicity of nigericin and its fluorinated analogues on wild-type mouse embryonic fibroblast cell line (WTMEFs). (A) Effect of molecules upon 2 hour treatment and (F) effect of molecules upon 24 hour treatment. (B) and (G) are WTMEFs untreated cells and (C–E) and (H–J) cell morphology, upon 2 h and 24 h treatment with 10 μM nigericin (DAS29), fluorinated analogue 3 (NIG-3) and fluorinated analogue 5 (NIG-5) respectively.

Chemical modification is aimed to improve the overall efficacy of molecules by eliminating or alleviating toxicity and adverse side effects. Nigericin and other ionophoric secondary metabolites are known for their potent activity; however, the associated higher toxicity reduces its overall efficacy, thus making it toxic in clinical setups. Here, nigericin produced by *Streptomyces* sp. DASNCL-29 was compared with the fluorinated analogues NIG-3 and NIG-5 for *in vitro* cytotoxicity on wild type mouse embryonic fibroblast cell line (WTMEFs) viability using 3-(4,5-dimethylthiazol-2-yl)-2,5-diphenyltetrazolium bromide assay (MTT assay)^36^ (Fig. 2). In the cytotoxicity experiment, nigericin showed an IC_50_ of ∼10 μM in 2 h and 24 h treatment. However, the IC_50_ for the fluorinated analogues NIG-3 and NIG-5 were higher than 100 μM (highest treated concentration) for 2 h treatment, and it was 66.87 and 59.76 μM for 24 h treatment, respectively. The effect of nigericin and fluorinated analogues NIG-3 and NIG-5 on wild-type mouse embryonic fibroblast cell line along with IC_50_ values (μM) for 2 h and 24 h experiments (*n* = 3), are provided in Fig. 2A–J. Fluorinated analogues of nigericin showed ten-fold lesser toxicity than the parent molecule together with improved efficacy. High efficacy and low toxicity, which is attributed to an excellent therapeutic index of nigericin fluorinated analogues, makes them a noble clinical candidate.

## Experimental

### Fermentation, extraction and purification of bioactive metabolites

Seven-day grown log phase culture was used for fermentation. Inoculum development was carried-out using liquid cultivation medium (LCM) (g L^−1^) soy-meal 20.0, mannitol 20.0 and glucose 4.0; 10% inoculum was used from LCM to inoculate into metabolite production medium 1 containing (g L^−1^) starch 15.0, yeast extract 4.0, K_2_HPO_4_ 1.0 and MgSO_4_·7H_2_O 0.5. Ten-litre lab-scale fermenter (Eppendorf BioFlow® CelliGen® 115) was used, and intermittent samples were collected to verify for any contamination and production of the bioactive metabolite by performing antimicrobial activity in the crude sample against the test organism. Fermentation broth together with cells was harvested into a baffled extraction vessel. The harvested broth was mixed with an equal amount of ethyl acetate and extracted three times using mechanical mixing. Ethyl acetate extract was then separated from the aqueous phase using centrifugation (2500 rpm for 5 min) and evaporated to concentrate using a rotary evaporator. The crude extract was fractionated using a silica gel (Merk 100–200) gravity column under the gradient solvent system of dichloromethane (DCM) and methanol (MeOH). Thin-layer chromatography (TLC Merk silica gel F-254) and visualisation on TLC was monitored by *p*-anisaldehyde. The fraction containing activity was footprinted by bioactivity assays and purified under the gradient solvent system of petroleum ether and ethyl acetate. The purity of the bioactive compound obtained was analysed by ^1^H NMR and thin-layer chromatography (TLC Merk silica gel F-254). ^1^H NMR (400 Hz) and ^13^C NMR (100 Hz) spectra were recorded at 400 MHz on Bruker Avance 300 ultrashield NMR instrument. All chemical shifts (*δ*) were reported in ppm downfield from trimethylsilane (0.007) as an internal standard in CDCl_3_ (7.27 ppm for ^1^H and 77.00 ^13^C) purchased from Merck-Sigma. Spin multiplets were reported as s (singlet), d (doublet), t (triplet), q (quartet) and m (multiplets). The coupling constant, *j*, is reported in Hz. X-ray intensity data measurements of nigericin crystal were performed on a Bruker D8 VENTURE Kappa Duo PHOTON II CPAD diffractometer equipped with incoatech multilayer mirrors optics. The intensity measurements were performed using Mo micro-focus sealed tube diffraction source (MoKα = 0.71073 Å) at 100(2) K temperature. The X-ray data collection was monitored by the APEX3 program (Bruker, 2016), whereas the chromatographic analysis was performed on 1260 Infinity II Prime LC (800 bar) equipped with Infinity Lab Poroshell 120 EC-C18. 2.1 × 150 mm, 1.9 μm (Agilent, USA) column. The column temperature was maintained at 40 °C with a flow rate of 0.3 ml min^−1^. The standard ten ppm of nigericin (Sigma, USA) and bioactive compound extracted from the *Streptomyces* sp. DASNCL-29 were separated using a 20 min linear gradient of 100% MS grade water (solvent A) and 100% MS grade acetonitrile (solvent B) containing 0.1% formic acid. The LC eluent was introduced into the mass spectrometer (Agilent 6530 Accurate-Mass Q-TOF/MS) with dual ESI as an ionisation source. Q-TOF tuning and mass calibrations were performed using ESI-L low concentration tuning mixture (Part No.: G1969-85000, Agilent USA). The MS system was operated using the positive-ion (ESI+) mode. Conditions of positive-ion analysis were as follows: nitrogen gas as the nebulising gas; the gas temperature was 352 °C, drying gas flow was 10 L min^−1^, nebuliser of 35 psi, fragmented voltage of 120 V, and *V*_Cap_ of 4 kV. The MS data were acquired in the mass range of 100–1300 *m*/*z*.

### Chemical modification by fluorine addition

To generate fluorinated analogues of nigericin, first, the acid group of the molecule “1” was converted into the acid chloride, which is an intermediate compound “2” using thionyl chloride in DCM at 50 °C for 6 h. Furthermore, we performed the esterification of the intermediate compound “2” using substituted phenol and aniline to get the final fluorinated synthetic analogues “3” complete flow of chemical modification, which is presented in Scheme 1.

### The bioactive potential of the purified compounds

Purified molecules together with chemically modified analogues were tested against Gram-positive (*S. aureus* ATCC 9144, *S. epidermidis* ATCC 12228, *B. subtilis* ATCC 6051, *B. cereus* ATCC 10876) and Gram-negative (*E. coli* ATCC 8739, *P. aeruginosa* ATCC 19154, *K. pneumoniae* ATCC 700603, *S. typhi* ATCC 23564) test organisms by spread plate-disc diffusion assay method (Wiegand *et al.*, 2008). Moreover, 20 μl from 100 μg ml^−1^ stock concentration was used per disc for each test compound, and the plates were subjected to incubation at 37 °C for 24 h. After the incubation zone of inhibition was measured, the minimum inhibitory concentration (MIC) was determined for Gram-positive strain *S. aureus* ATCC 9144 and Gram-negative strain *E. coli* ATCC 8739 using the micro-dilution method in 96 well plates. Furthermore, 20.0 μl from 1.0 mg ml^−1^ stock solution was mixed with test strain (5 × 10^5^ cfu ml^−1^) to obtain 66.0 μg ml^−1^ concentration and serially diluted in wells to get a concentration of 33.0 μg ml^−1^, 16.5 μg ml^−1^ and up to 2.0 ng ml^−1^.

Micro titre plates were incubated in a plate shaker incubator at 37 °C with 150 rpm for 24 h. After 24 h of incubation, the plates were observed for the presence of growth and inhibitions. Morphologies of treated bacterial cells of *S. aureus* ATCC 9144 and *E. coli* ATCC 8739 were compared with the control cells. The effect of purified molecule and its fluorinated analogs on bacterial cells was observed under transmission electron microscopy (TEM). Cells were fixed following the standard protocol for observation in TEM.^27^ In brief, the bacterial cells were stabilised by treatment with glutaraldehyde, followed by osmium tetroxide. Later, the bacterial cells were dehydrated by a series of ethanol wash, and 10 μl from 100% ethanol was loaded on to the copper grid and air-dried. TEM images were recorded at 200 kV using a Tecnai field emission electron microscope.

Furthermore, activity against *Plasmodium falciparum*, a malarial parasite, was carried out by SYBR green staining-based parasitemia determination, as previously reported (Martin *et al.* 2004). The assays were set up in a 96-well plate using 200 μl of culture per well at 2.0% parasitemia. The stock solution of the purified molecules, together with standard nigericin (Sigma-Aldrich) was prepared in DMSO, such that the final concentration of DMSO in the assay did not exceed 1.0%. For comparison, 1% of DMSO treated cultures were included as controls. For dose assays, a two-fold dilution series of the inhibitors from 10.0 μM to 0.01 μM were used. The standard antimalarial drug, chloroquine, was included as positive controls at concentration of 1.0 μm.

### Cytotoxicity

Wild-type mouse fibroblast cell line (WTMEFs) was used to assess the toxicity associated with nigericin and its fluorinated analogues. WTMEFs cell line was seeded in 96-well plates at a density of 5000 cells per well in 100 μl DMEM media containing 10% FBS. The cell lines were incubated for 24 h in a CO_2_ incubator at 37 °C. After 24 h, the drug was added at a concentration of 100, 80, 40, 20, 10 and 1.0 μM and incubated for 2 h and 24 h. MTT assay was carried out to verify the cell viability after 2 h and 24 h of drug treatment in respective plates. All drug concentration was used in triplicate (*n* = 3).

## Conclusion

For the first time, a high yield of nigericin molecule is reported from *Streptomyces* sp. DASNCL-29 as the main product of the fermentation. Furthermore, we identified that the producer strains have the nigericin biosynthetic gene cluster, together with an associated additional transport-related gene from the sequenced genome of DASNCL-29, which co-relates the higher yield of the molecule and was not reported earlier. Optimised fermentative protocol resulted in higher yields of nigericin from *Streptomyces* DASNCL-29, which was ∼500 mg L^−1^.

For the first time, isolated nigericin molecules exhibited a monoclinic and orthorhombic spatial crystal arrangement, which revealed its polymorphic nature. However, the synthesis of fluorinated chemical derivative showed potent antibacterial activity against Gram-positive and Gram-negative pathogens. Moreover, for the first time, chemical modification of nigericin analogues showed activity against Gram-negative organisms that will help in treating the Gram-negative infections, and the toxicity of fluorinated analogues was found to be tenfold lesser than the parent nigericin. These findings suggest that the multidimensional potential of nigericin as a drug candidate has been neglected since its discovery. The fluorinated analogues of nigericin showed a promising way to use against Gram-negative pathogens, which are a major threat to healthcare due to the emergence of antimicrobial resistance.

## Conflicts of interest

There are no conflicts to declare.

## Supplementary Material

RA-010-D0RA05137C-s001

RA-010-D0RA05137C-s002
