# Supplementary material for: Approach to nigericin derivatives and their therapeutic potential
Source: RSC Adv. 2020 Nov 26;10(70):43085–91. doi: 10.1039/d0ra05137c (PMC9058090; doi:10.1039/d0ra05137c)
Supplement: RA-010-D0RA05137C-s001 [file RA-010-D0RA05137C-s001.pdf]

Electronic Supplementary Material (ESI) for RSC Advances.
This journal is © The Royal Society of Chemistry 2020

ELECTRONIC SUPPLEMENTARY MATERIALS

Approach to nigericin derivatives and their therapeutic potentials

Amit Kumar Sahu[1,2,3], Madhukar S Said[1,4], Tejashri Hingamire[1,3], Manan B Shah[2], Megha Gaur[6], Abujunaid Khan[1,2,3], Dhanasekaran Shanmugam[1,3], Vitthal T Barvkar[5], Mahesh S Dharne[1,2,3], Atul A Bharde[6], Syed G Dastager[1,2,3*]

[1] Academy of Scientific and Innovative Research (AcSIR), CSIR National Chemical Laboratory Pune-411008, India.

[2] National Collection of Industrial Microorganisms (NCIM), CSIR National Chemical Laboratory Pune-411008, India.

[3] Biochemical Sciences Division, CSIR National Chemical Laboratory Pune-411008, India.

[4] Organic Chemistry Division, CSIR National Chemical Laboratory Pune-411008, India.

[5] Department of Botany, Savitribai Phule-Pune University, Pune-411007, India.

[6] Department of Microbiology, Savitribai Phule-Pune University, Pune-411007, India.

* Corresponding author: Dr. Syed G Dastager (Email: sg.dastager@ncl.res.in)

Address: NCIM Resource Centre, CSIR National Chemical Laboratory, Pune, India 411008.

CONTENTS

S.No.	Content	Page no.
1.	Isolation and Identification of bioactive compound producer strain	S3
2.	Phylogenetic tree, *In-Silico* DNA-DNA hybridisation and MALDI TOF MS-based strain comparison and biochemical characterisation	S5-S11
3.	Biosynthetic gene cluster	S12
4.	Submerged Fermentation Upstream and Downstream Process parameter	S13
5.	NMR Spectrum , ^{1}H, ^{13}C, DEPT, HMBC, HSQC, NOSY, COSY	S14-16
6.	X-ray crystallography and HPLC-QTOF-MS Spectrum of purified metabolite and standard.	S17-19
7.	Structure of nigericin and is analogues, NMR data	S20-S22
8.	Bioactive potentials, antibacterial and antimalarial	S23-25

Isolation and Identification of Bioactive compound producer strain.

In this study, a novel indigenous species of *Streptomyces* strain DASNCL-29 was isolated from a plant-associated soil sample collected from Unkeshwar, Maharashtra, India (suppli.Fig.1). Qualitative screening for bioactivity was carried out. The fermentative broth of the isolates grown in different metabolite production medium for seven days at 150 rpm kept at 28°C. Isolated strains with bioactive potentials were further characterised by 16S rRNA gene sequencing and whole genome. Sequencing was performed on Sanger sequencing platform (Applied Biosystem® 3500xL Genetic Analyser). The potent strain was characterised by whole genome sequencing using Illumina HiSeq and Oxford Nanopore MinION (Oxford Nanopore Technologies, Oxford, UK) according to nanopore protocol for 1D native barcoding genomic DNA (EXP-NBD103) and 1D genomic DNA by ligation sequencing kit SQK-LSK 109 as per the recommendation of the manufacturer. Phylogenetic relatedness of *Streptomyces sp.* DASNCL29 was studied using 16S rRNA gene sequence blast in Ez-Bio cloud database (http://www.ezbiocloud.net)[1] (Supplementary Figure. 2)

MALDI-TOF MS spectra based strain comparison was done with closest strains. To generate MS spectra (Supplementary figure 3), a pure colony of the bacteria was applied as a thin film on a target plate using a toothpick and dried for 1 min at room temperature, following 0.5µl of 70% formic acid was mixed with the sample and by 0.5µl of acetonitrile, and the resultant mixture was dried at room temperature for 10min. Finally, 1.5µl of the matrix solution (comprising a saturated a-cyano-4-hydrocinnamic acid in 50% acetonitrile HPLC grade and 2.5% trifluoroacetic acid) was applied onto the spot.[2-4] MS spectra were generated on Bruker Biotyper MALDI-TOF MS system. The

biochemical characterisation and comparison were made based on biochemical test data obtained from BCL card and GP card of Vitek 2 Compact (BioMérieux India) and API Zym kit (BioMérieux India) (Supplementary Table. 1, 2 and 3). For identification and characterisation of the biosynthetic gene cluster in *Streptomyces* sp. DASNCL-29 assembled genome was configured using ViroBlast tool.[5] This sequence was further submitted to the antiSMASH webserver[6] to understand the biosynthetic gene cluster (Supplementary Figure 4 and Supplementary Table 4).

S_Figure 1: *Streptomyces* sp. DASNCL29 on ISP-2 medium. A) Aerial mycelium B) Substrate mycelium C)Gram's staining and D) Scanning Electron Micrograph.

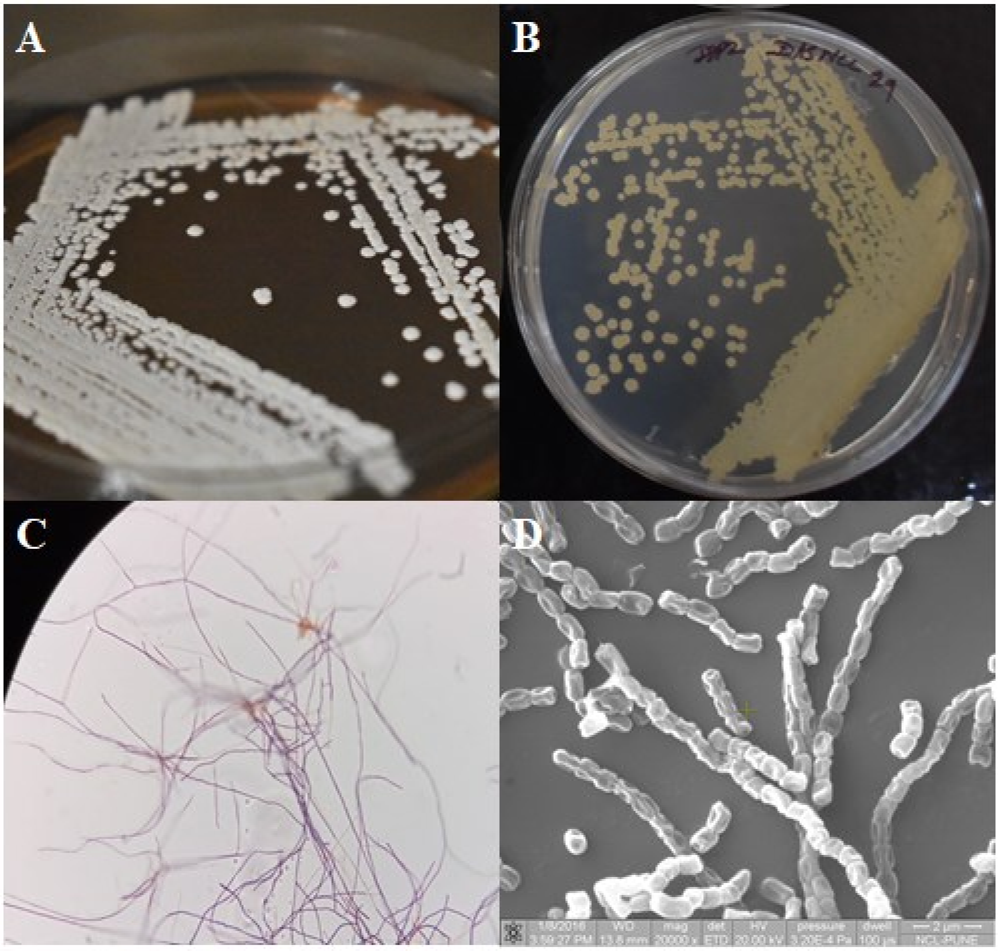

Phylogenetic tree, In-silico DNA-DNA hybridisation and MALDI TOF MS-based strain comparison

The tree is drawn to scale, with branch lengths in the same units as those of the evolutionary distances used to infer the phylogenetic tree.

S_Figure 2: Evolutionary relationships of *Streptomyces* sp. DASNCL29

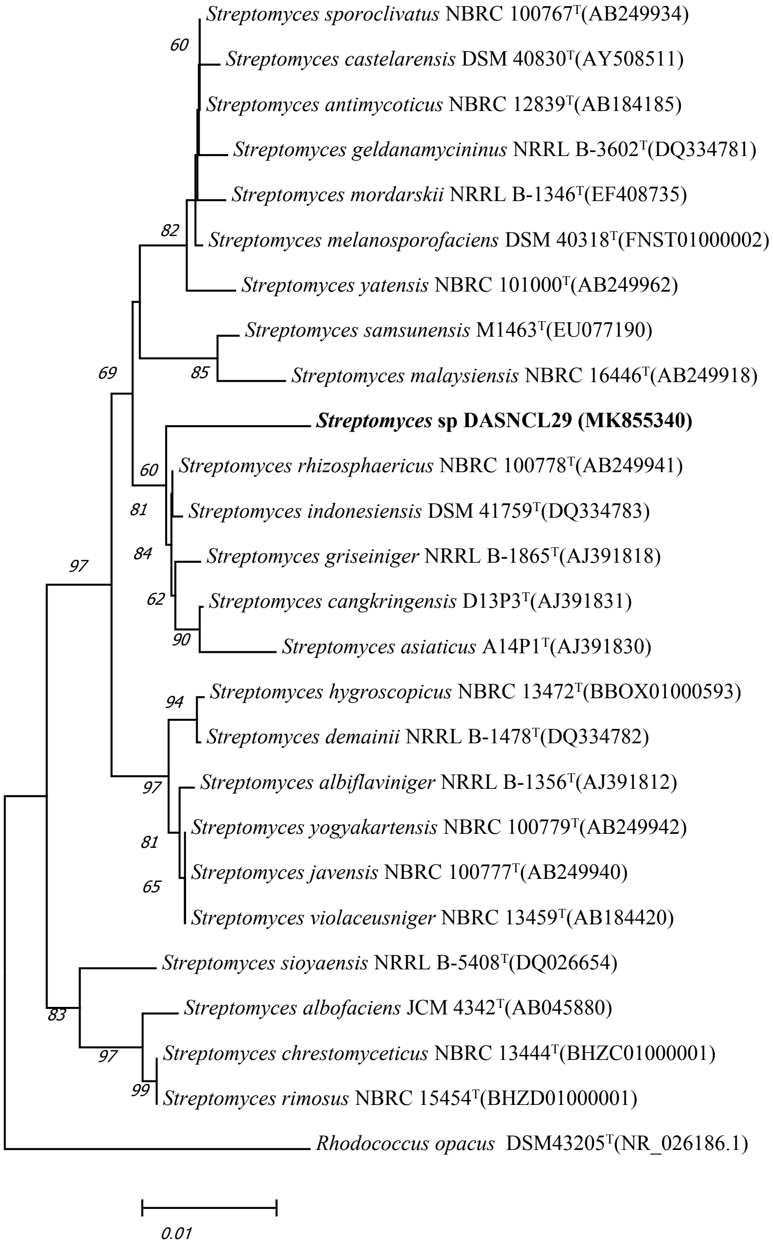

The evolutionary distances were computed using the maximum composite likelihood method [Tamura et al. 2004] and are in the units of the number of base substitutions per site. The analysis involved 26 nucleotide sequences. All positions containing gaps and missing data were eliminated. There were a total of 1369 nucleotides in the final dataset. Evolutionary analyses were conducted in MEGA-7 (Felsenstein *et al* 1985; Kumar *et al.* 2016).

S_Figure 3: A) Similarity between the genome of *Streptomyces* sp. strain DASNCL29 against the genomes of reference strains by in-silico genome to genome comparison. **B)** Comparison of the MALDI-TOF mass spectra of *Streptomyces* sp DASNCL29 with its phylogenetic neighbouring strains.

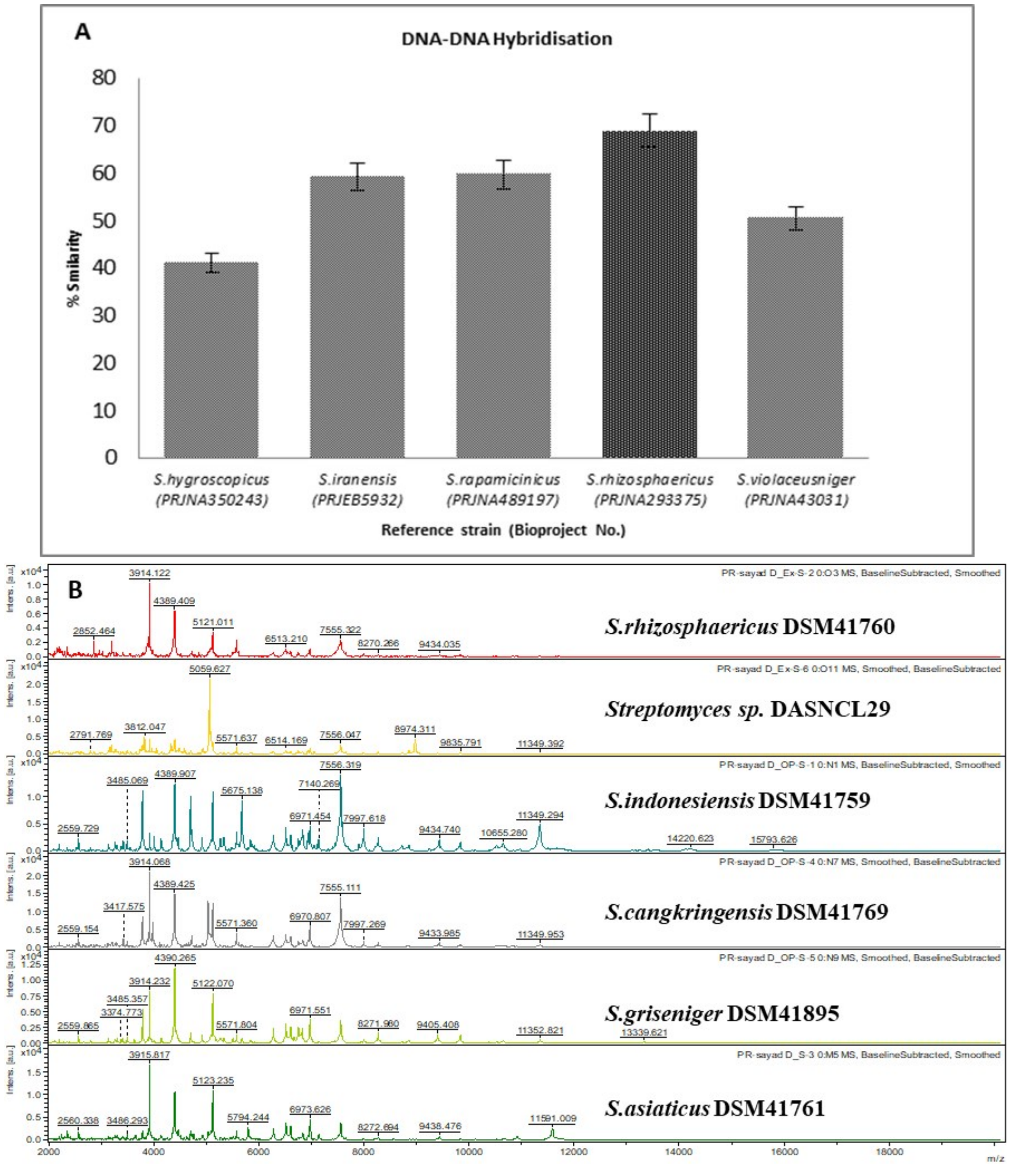

S_Table 1: Biochemical characterisation of *Streptomyces* sp. DASNCL29 and neighbouring strains using BCL card of VITEK 2 COMPACT

TEST	Amount per well	*Streptomyces* sp. DASNCL29	*S.indonesiensis* DSM41759	*S.rhizosphaericus* DSM41760	*S.congkringensis* DSM41769	*S.griseniger* DSM41895
1 BETA-XYLOSIDASE	0.0324 mg	+	+	-	+	-
3 L-Lysine-ARYLAMIDASE	0.0228 mg	+	+	+	+	+
4 L-Aspartate ARYLAMIDASE	0.024 mg	+	-	-	+	+
5 Leucine ARYLAMIDASE	0.0234 mg	+	+	+	+	+
7 Phenylalanine ARYLAMIDASE	0.0264 mg	+	+	+	+	+
8 L-Proline ARYLAMIDASE	0.0234 mg	+	+	+	+	+
9 BETA-GALACTOSIDASE	0.036 mg	+	-	-	+	+
10 L-Pyrrolidonyl-ARYLAMIDASE	0.018 mg	-	-	-	-	-
11 ALPHA-GALACTOSIDASE	0.036 mg	+	-	-	+	+
12 Alanine ARYLAMIDASE	0.0222 mg	+	+	+	+	+
13 Tyrosine ARYLAMIDASE	0.0282 mg	+	+	+	+	+
14 BETA-N-ACETYL-GLUCOSAMINIDASE	0.0408 mg	-	-	-	-	+
15 Ala-Phe-Pro ARYLAMIDASE	0.0384 mg	+	+	+	+	+
18 CYCLODEXTRIN	0.3 mg	-	-	-	-	-
19 D-GALACTOSE	0.3 mg	-	-	-	-	-
21 GLYCOGEN	0.1875 mg	-	+	-	+	-
22 myo-INOSITOL	0.3 mg	-	-	-	-	-
24 METHYL-A-D-GLUCOPYRANOSIDE	0.3 mg	-	-	+	+	-
25 ELLMAN	0.03 mg	-	-	-	-	+
26 METHYL-D-XYLOSIDE	0.3 mg	-	-	+	-	-
27 ALPHA-MANNOSIDASE	0.036 mg	+	+	-	+	+
29 MALTOTRIOSE	0.3 mg	-	+	+	-	-

30 Glycine ARYLAMIDASE	0.012 mg	+	+	-	+	-
31 D-MANNITOL	0.3 mg	-	-	-	+	-
32 D-MANNOSE	0.3 mg	-	-	-	+	-
34 D-MELEZITOSE	0.3 mg	-	-	+	+	+
36 N-ACETYL-D-GLUCOSAMINE	0.3 mg	-	-	+	-	-
37 PALATINOSE	0.3 mg	-	+	+	+	-
39 L-RHAMNOSE	0.3 mg	-	+	-	-	-
41 BETA-GLUCOSIDASE	0.036 mg	-	+	-	-	-
43 BETA-MANNOSIDASE	0.036 mg	-	-	-	+	-
44 PHOSPHORYL CHOLINE	0.0366 mg	+	+	+	+	+
45 PYRUVATE	0.15 mg	-	-	-	-	+
46 ALPHA-GLUCOSIDASE	0.036 mg	-	+	-	-	-
47 D-TAGATOSE	0.3 mg	-	-	-	-	-
48 D-TREHALOSE	0.3 mg	-	-	+	-	-
50 INULIN	0.12 mg	-	-	-	+	+
53 D-GLUCOSE	0.3 mg	-	-	-	-	-
54 D-RIBOSE	0.3 mg	-	-	-	-	-
56 PUTRESCINE assimilation	0.201 mg	-	-	-	+	-
58 GROWTH IN 6.5% NaCl NaCl 6.5%	1.95 mg	-	-	-	-	-
59 KANAMYCIN RESISTANCE	0.006 mg	-	-	-	-	-
60 OLEANDOMYCIN RESISTANCE	0.003 mg	-	-	-	-	-
61 ESCULIN hydrolyse	0.0225 mg	-	+	-	+	-
62 TETRAZOLIUM RED	0.0189 mg	-	-	-	-	-
63 POLYMIXIN_B RESISTANCE	0.00093 mg	-	-	-	-	-

Key + = Positive, - = Negative

S_Table 2: Biochemical characterisation of *Streptomyces* sp. DASNCL29 and neighbouring strains using GP card of VITEK 2 COMPACT

**TEST**	**Amount per well**	***Streptomyces* sp. DASNCL29**	***S.indonesiensis* DSM41759**	***S.rhizosphaericus* DSM41760**	***S.congkringensis* DSM41769**	***S.griseniger* DSM41895**
2 D-AMYGDALIN	0.1875 mg	-	+	+	-	-
4 PHOSPHATIDYLINOSITOL PHOSPHOLIPASE C PIPLC	0.015 mg	+	+	+	+	+
5 D-XYLOSE dXYL	0.3 mg	-	-	+	-	-
8 ARGININE DIHYDROLASE 1 ADH1	0.111 mg	-	+	-	+	+
9 BETA-GALACTOSIDASE BGAL	0.036 mg	-	+	-	-	-
11 ALPHA-GLUCOSIDASE AGLU	0.036 mg	-	+	-	+	-
13 Ala-Phe-Pro ARYLAMIDASE APPA	0.0384 mg	+	+	+	+	+
14 CYCLODEXTRIN CDEX	0.3 mg	-	-	-	-	-
15 L-Aspartate ARYLAMIDASE AspA	0.024 mg	-	-	-	-	-
16 BETA GALACTOPYRANOSIDASE BGAR	0.00204 mg	+	+	-	+	+
17 ALPHA-MANNOSIDASE AMAN	0.036 mg	+	+	-	+	+
19 PHOSPHATASE PHOS	0.0504 mg	-	+	-	-	-
20 Leucine ARYLAMIDASE LeuA	0.0234 mg	+	+	+	+	+
23 L-Proline ARYLAMIDASE ProA	0.0234 mg	-	+	+	+	-
24 BETA GLUCURONIDASE BGURr	0.0018 mg	-	-	-	-	-
25 ALPHA-GALACTOSIDASE AGAL	0.036 mg	+	-	-	+	+
26 L-Pyrrolydonyl-ARYLAMIDASE PyrA	0.018 mg	-	-	-	-	-
27 BETA-GLUCURONIDASE BGUR	0.0378 mg	-	-	-	-	-
28 Alanine ARYLAMIDASE AlaA	0.0216 mg	+	+	+	+	+
29 Tyrosine ARYLAMIDASE TyrA	0.0276 mg	+	+	+	+	+
30 D-SORBITOL dSOR	0.1875 mg	-	-	-	-	-
31 UREASE URE	0.15 mg	+	-	-	+	+

32 POLYMIXIN B RESISTANCE POLYB	0.00093 mg	-	-	-	-	-
37 D-GALACTOSE dGAL	0.3 mg	-	-	-	-	-
38 D-RIBOSE dRIB	0.3 mg	-	-	-	-	-
39 L-LACTATE alkalinization ILATk	0.15 mg	-	-	-	+	-
42 LACTOSE LAC	0.96 mg	-	-	-	-	-
44 N-ACETYL-D-GLUCOSAMINE NAG	0.3 mg	-	-	-	-	-
45 D-MALTOSE dMAL	0.3 mg	-	-	-	-	-
46 BACITRACIN RESISTANCE BACI	0.0006 mg	-	-	-	-	-
47 NOVOBIOCIN RESISTANCE NOVO	0.000075 mg	-	-	-	-	-
50 GROWTH IN 6.5% NaCl NC6.5	1.68 mg	-	-	-	-	-
52 D-MANNITOL dMAN	0.1875 mg	-	-	-	-	-
53 D-MANNOSE dMNE	0.3 mg	-	-	+	-	-
54 METHYL-B-D-GLUCOPYRANOSIDE MBdG	0.3 mg	-	-	+	-	-
56 PULLULAN PUL	0.3 mg	-	-	-	-	-
57 D-RAFFINOSE dRAF	0.3 mg	-	-	+	-	-
58 O/129 RESISTANCE (comp.vibrio.) O129R	0.0084 mg	-	-	-	-	-
59 SALICIN SAL	0.3 mg	-	-	+	-	-
60 SACCHAROSE/SUCROSE SAC	0.3 mg	-	-	+	-	-
62 D-TREHALOSE dTRE	0.3 mg	-	-	-	-	-
63 ARGININE DIHYDROLASE 2 ADH2s	0.27 mg	+	-	-	+	+
64 OPTOCHIN RESISTANCE OPTO	0.000399 mg	-	-	-	-	-

Key + = Positive, - = Negative

S_Table 3: Biochemical characterisation of *Streptomyces* sp. DASNCL29 and neighbouring strains using APi Zym kit.

Enzyme Assayed	*Streptomyces* sp. DASNCL29	*S.indonesiensis* DSM41759	*S.rhizosphaericus* DSM41760	*S.congkringensis* DSM41769	*S.griseniger* DSM41895
Control	-	-	-	-	-
Alkaline Phosphatase	+	+	+	+	+
EsteraseC4)	-	-	-	-	-
Esterase Lipase (C8)	-	-	-	-	-
Lipase (C14)	-	-	-	-	-
Leucine arylamidase	+	+	+	+	+
Valine arylamidase	+	-	-	+	+
Cystine arylamidase	-	-	-	-	-
Trypsin	+	-	+	+	+
α-chymotrypsin	+	-	+	+	+
Acid Phosphatase	+	+	+	+	+
Napthol phosphohydrolase	+	+	+	+	+
α-galactosidase	-	-	-	-	-
β-galactosidase	+	-	-	-	-
β-glucuronidase	-	-	-	-	-
α-glucosidase	-	+	-	-	-
β-glucosidase	-	+	+	+	+
N-acetyl β-glucosaminidase	+	-	+	+	+
α-mannosidase	-	+	-	-	-
α-fucosidase	-	-	-	-	-

Key + = Positive, - = Negative

S_Table 4: Biosynthetic Gene cluster present in *Streptomyces* sp. DASNCL-29

Gene Cluster/Type	From	To	Similarity
Azalomycin F/ T1PKS*	596111	732551	100 %
Nigericin/ T1PKS*	896644	1072478	100 %
Ectoine/ Ectoine	1921521	1931925	100 %
2-methylisoborneol/ terpene	3257140	3277203	100 %
Pristinol/ terpene	3751476	3771260	100 %
Hygrocin/ T1PKS*	4186929	4268985	93 %
Echosides/ NRPS**	145596	185955	100 %
Desferrioxamine B/ Siderophore	701056	712843	100 %
Geosmin/ Terpene	1821630	1843975	100%
*Type 1 Polyketide Synthases **Nonribosomal peptide			

S_figure 4: Organization of Nigericin Biosynthetic Gene Cluster (NBGC), along with its location on the genome of *Streptomyces* sp. DASNCL29. Circular genome depiction was made using Cgview (Stothard *et.al* 2005) and Gview (Aaron *et al*. 2010). Modular Polyketide synthase gene (Red), regulatory genes (Purple), auxillary PKS genes (light blue) and Transporter gene (Yellow, highlighted in light blue). Cyclase gene (green), Cytochrome P450 hydroxylase (Brown) and FAD dependant epoxidase is shown in blue.

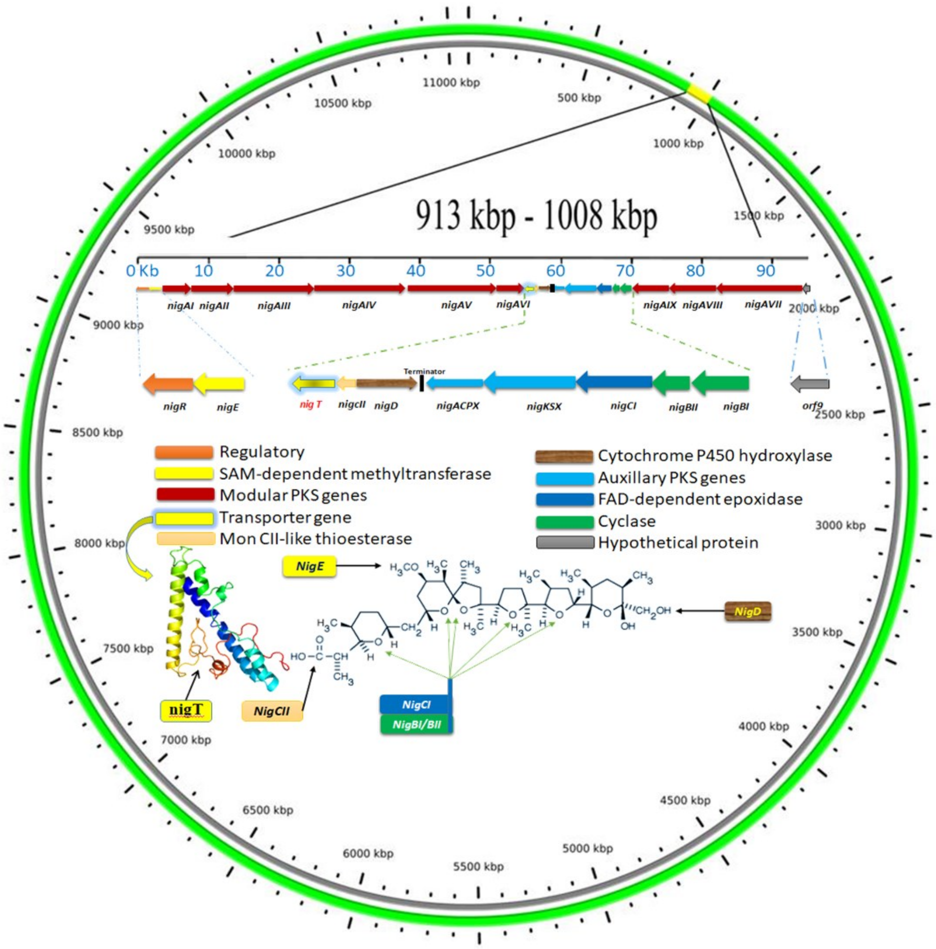

Submerged Fermentation Upstream and Downstream Process for production of bioactive metabolites

Fermentation optimization: Fermentation optimization for production of Nigericin was done by using different media composition. For optimisation the physiological parameters like temperature, pH, volume and DO was kept constant. Inoculum for all sets of production media was same. During optimisation the production of Nigericin was assessed by checking the antibacterial efficacy of crude extract by dilution assay. Production media 1 was found to the best for higher yield of Nigericin.

S_Table 5: Upstream process parameter for the production of Nigericin from *Streptomyces* sp. DASNCL-29.

**A. Different media composition used for Optimization of Nigericin Production**		
	**Media**	**Composition**
LCM	Liquid Cultivation	Soy-meal 20g/l, mannitol 20g/l, and glucose 4g/l
Production media	1	Starch 15g/l, Yeast extract 4g/l, K_2HPO_4 1g/l, $MgSO_4.7H_2O$ 0.5g/l
	2	Glucose 15g/l, Soyameal 5g/l, Cornsteep liquor 5g/l, $CaCO_3$ 2g/l, NaCl 5g/l
	3	Starch 10g/l, Yeast extract 2g/l, Glucose 10g/l, Glycerol 10g/l, CSL 2.5g/l, Peptone 2g/l, NaCl 1g/l, $CaCO_3$ 3g/l
	4	Glucose 4g/l, Yeast Extract 4g/l, Malt Extract 10.0g/l, $CaCO_3$ 2g/l
	5	Malt Extract 3g/l, Glucose 10g/l, Yeast Extract 3g/l, Peptone 5g/l
	6	Starch 10g/l, $CaCO_3$ 2g/l, K_2HPO_4 1g/l, $MgSO_4.7H_2O$ 1g/l, NaCl 1g/l
	7	Peptone 15g/l, Proteose peptone 5g/l, Ferric Ammonium Citrate 0.5g/l, K_2HPO_4 1g/l, Sodium thiosulfate 0.08g/l, Yeast extract 1g/l
	8	Tryptone 5g/l, Yeast extract 3g/l
**B. Upstream process parameter for the production of Nigericin**		
	**Parameter**	**Conditions/Values**
Inoculum Development	Volume	1.0 L (4x500ml flask with 250ml LCM)
	Temperature	28.0 ^{0}C
	Shaking	150 RPM (Shaking Incubator)
	Duration	6 Days
	pH	7.0± 0.3 (Not maintained during the process)
Metabolite Production	Volume	10 L (9 l media + 1l inoculum)
	Temperature	28.0 ^{0}C Maintained using Jacket control
	Agitation	150 Initial (Controlled loop to maintain DO) (Range 150 – 500 RPM using Ruston turbine impellers)
	Aeration	Compressed Sterile Air 3 LPM through macro sparger (Adjusted to maintain DO)
	Dissolved Oxygen (DO)	Initially saturated to 100%.<br>Maintained to 30 ± 5 %.
	Baffles	Yes
	Duration	6 Days
	pH	7.0± 0.3 (Not maintained during the process)

S_Figure 5: 1D and 2D NMR Spectroscopy of bioactive compound obtained from *Streptomyces* sp. DASNCL-29

^{1}H NMR Spectra

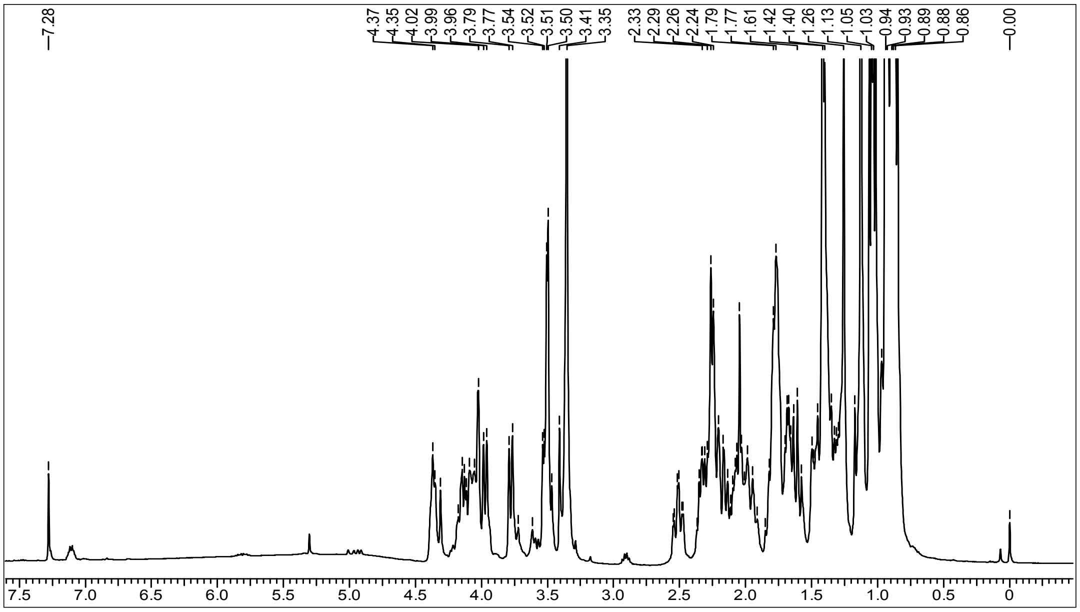

^{13}C NMR Spectra

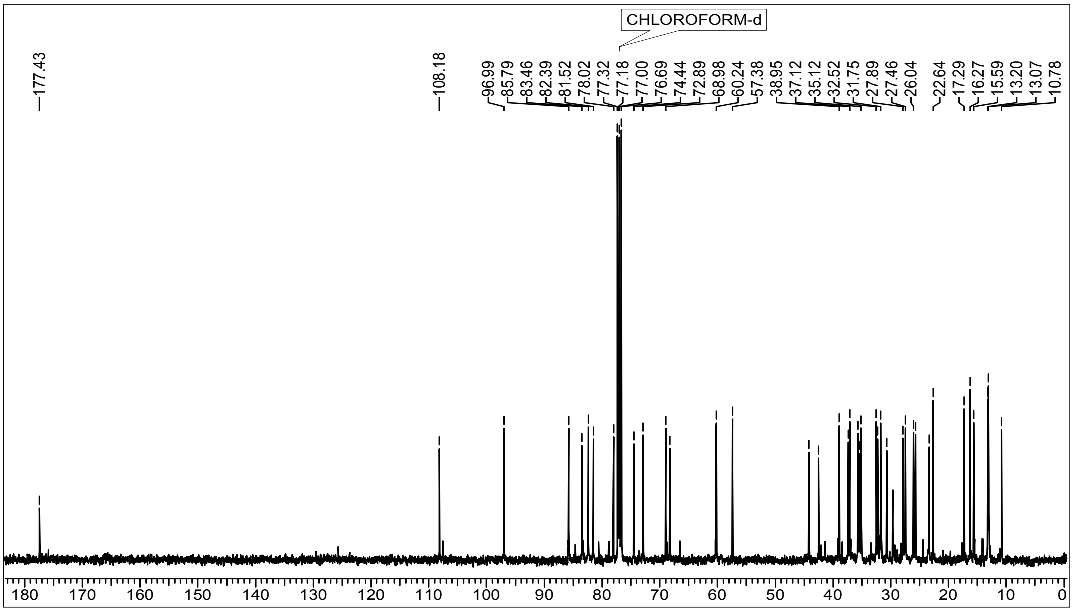

2D NMR of Bioactive compound obtained from *Streptomyces* sp. DASNCL29

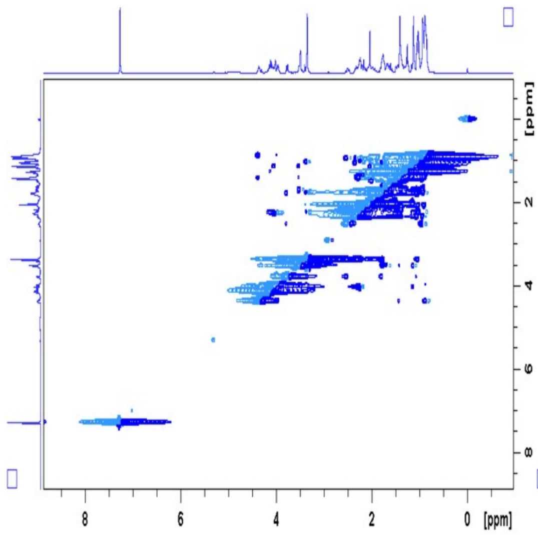

a: NOSY NMR SPECTRA

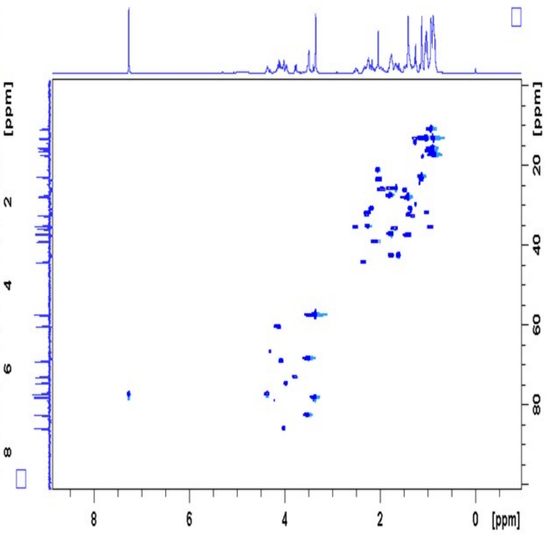

b: HSQC NMR SPECTRA

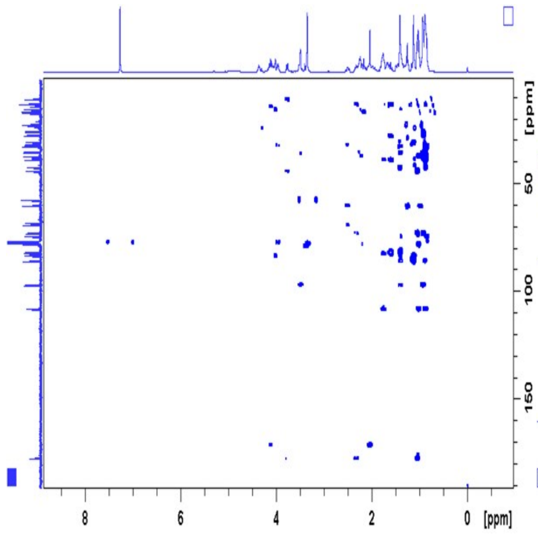

c: HMBC NMR SPECTRA

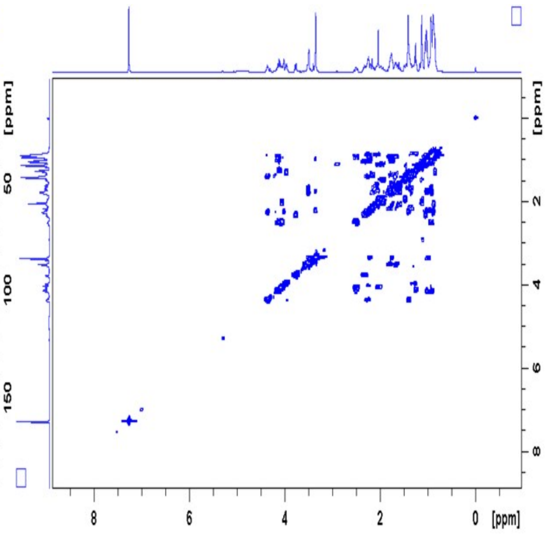

d: COSY NMR SPECTRA

S_Figure 6: X-ray crystallographic conformation of DASNCL29 derived nigericin in (A) Monoclinic, CCDC 1946291 (B) Orthorhombic CCDC 1946293.

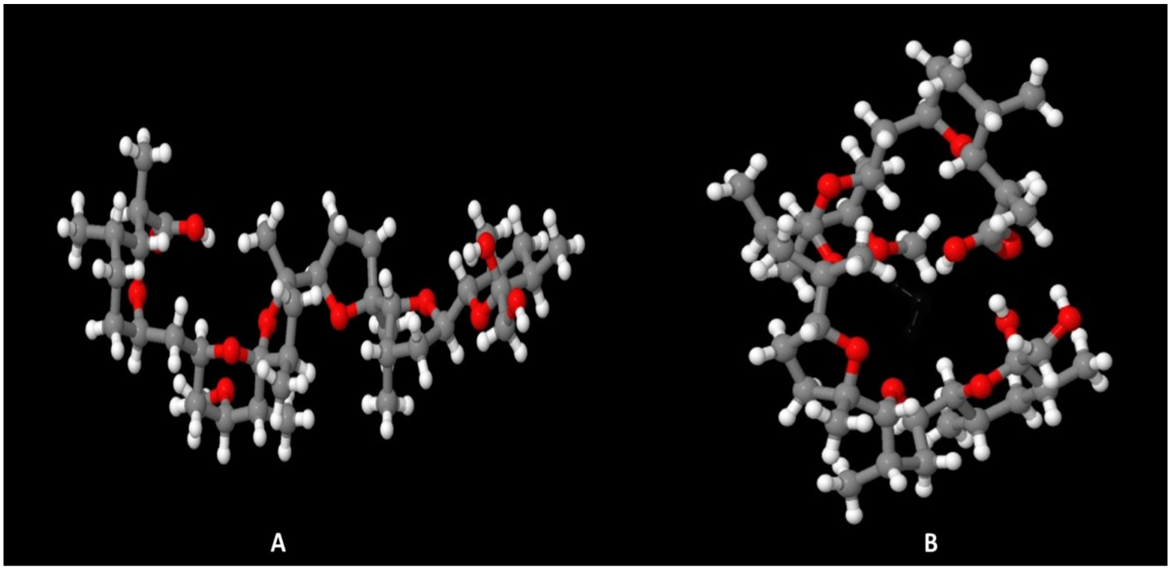

HPLC-QTOF MS-based analysis

The LC method started with 2% B for the first 0.3 min and increased to 30% in the next 2 min. The B percentage was increased from 30 to 45% till 7 min and further increased to 98% till 12 min and held for the next 3 min. The column was equilibrated to the initial ratio of solvents (98% A: 2% B) in the last 5 min. For accurate mass acquisition real-time mass correction was applied using Purine ([M+H]+ = 121.0508 Da) and HP-1221 ([M+H]+=1221.9906). The targeted MS/MS data were acquired with fixed collision energies; 10, 20 and 40 eV. The chromatograms were extracted with the help of Mass-Hunter Qualitative Workflow B.08.00 and compared with chromatogram and mass spectra of standard Nigericin. For MS/MS level confirmation, the exact mass of daughter ions from standard and sample Nigericin were compared.

S_Figure 7: HPLC-QTOF-MS Spectrum Bioactive compound

Chromatogram Overlay of Nigericin from DASNCL29(red) with Nigericin standard(black)

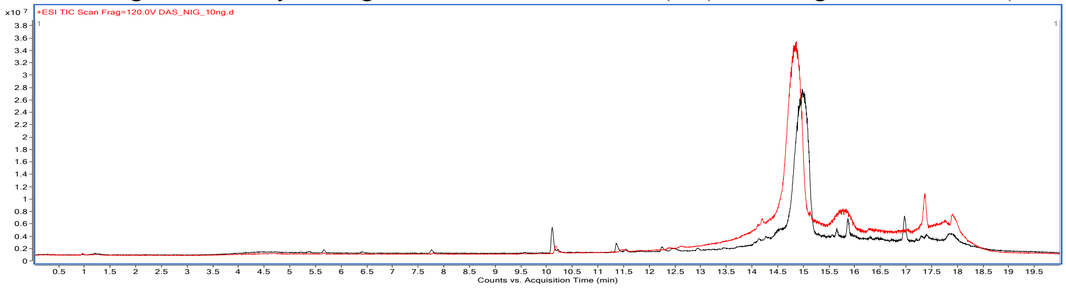

Extracted Ion chromatogram for Nigericin Sodium Adduct m/z 747.4654, Nigericin from DASNCL29(red) and Nigericin standard(black)

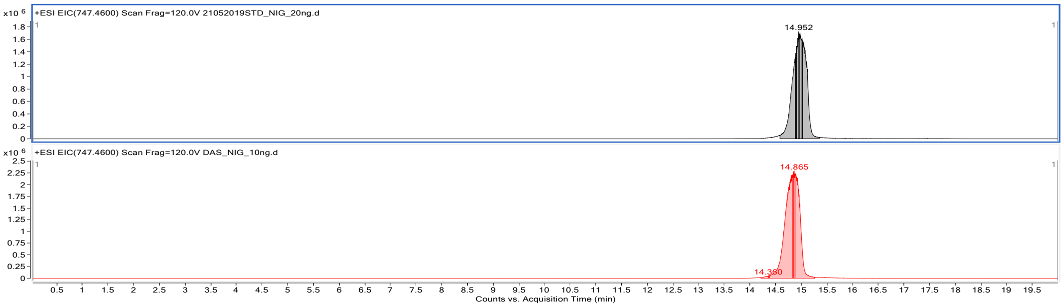

MS/MS fragments of Nigericin Adduct (M+NH4)+ (m/z: 742.50) at fragmentor voltage: 120V and CID 20eV, , Nigericin from DASNCL29(bottom) and Nigericin standard(top) (Matching fragments: 693.45, 675.44, 657.43, 639.42)

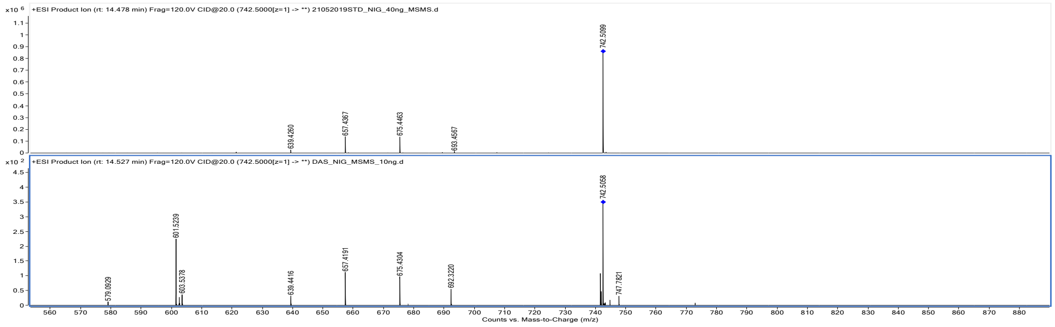

MS/MS fragments of Nigericin Adduct (M+Na)+ (m/z: 747.46) at Fragmentor voltage: 120V and CID 40eV, Nigericin from DASNCL29 (bottom) and Nigericin standard (top) (Matching fragment: 729.45)

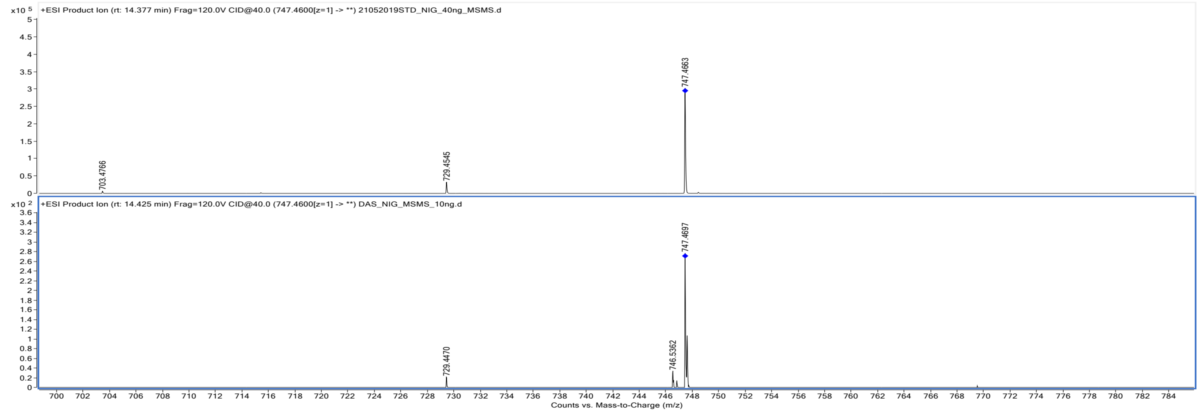

S_ figure.8. Structure of nigericin derived analogues.

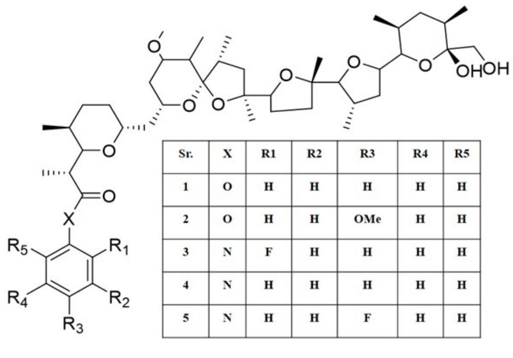

Sr.	X	R1	R2	R3	R4	R5
1	O	H	H	H	H	H
2	O	H	H	OMe	H	H
3	N	F	H	H	H	H
4	N	H	H	H	H	H
5	N	H	H	F	H	H

Nigericin:

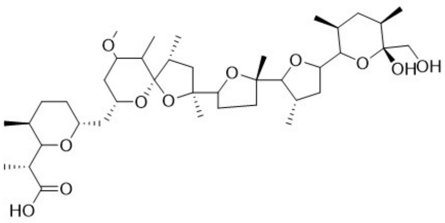

Analogue 1:

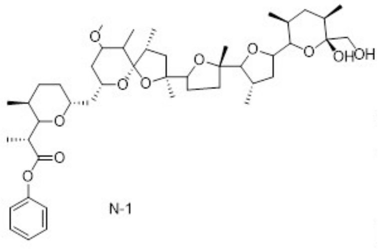

Analogue 2:

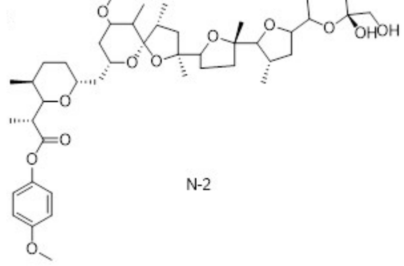

Analogue 3:

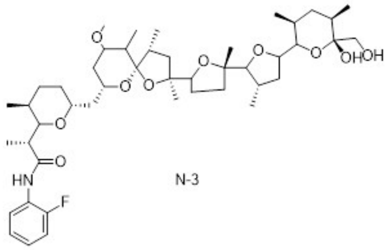

Analogue 4:

Analogue 5:

S_table 6: ^{13}C NMR spectra of nigericin and its bioactive analogues

Carbon No (δ_C)	Nigericin	Nigericin analogue-3	Nigericin analogue-5
1	177.4	174.4	174.3
13	108.2	108.5	108.7
29	97.0	97.0	97.1
21	85.8	85.7	85.5
20	83.4	83.6	83.6
14	82.4	82.7	82.3
17	81.5	81.4	81.2
11	78.0	78.8	78.5
25	77.3	77.5	77.6
24	74.4	74.6	74.4
3	72.9	72.4	72.4
7	69.0	69.0	69.1
30	68.3	68.4	68.2
9	60.2	61.3	61.2
40	57.4	57.4	57.7
2	44.2	44.4	44.7
15	42.5	42.5	42.8
14	38.9	38.4	38.4
27	37.4	37.4	37.7
28	37.1	37.1	37.1
12	35.7	35.7	35.5
8	35.3	35.6	35.7
22	35. 1	35.3	35.6
10	32.5	32.2	32.1
23	32.2	32.1	32.1
26	31.7	31.1	31.5
19	30.7	30.7	30.7
4	27.9	27.9	27.9
35	27.5	27.5	27.5
5	26.0	26.0	26.0
18	25.7	25.3	25.3
6	23.4	23.3	23.3
34	22.6	22.5	22.5
32	17.3	17.5	17.5
31	16.6	16.4	16.4
33	16.3	15.4	15.4
39	14.2	14.4	14.4

36	13.1	13.6	13.6
37	13.0	13.4	13.4
38	10.8	10.3	10.3
41		158.5 (d, *J* = 164 Hz)	161.5 (d, *J* = 165.3 Hz),
42		130.3 (d, *J* = 10.3 Hz)	131.2 (d, *J* = 8.9 Hz),
43		121.4	121.4,
44		123.8	116.5 (d, *J* = 19.2 Hz),
45		116.5 (d, *J* = 19.2 Hz)	
46		115.2	

Analog 1(N-1): ^{1}H NMR (400MHz, CHLOROFORM-d) 7.24(m, 2H) 6.91 (m, 1H), 6.84 (dd, 8.1, 2.2 Hz 2H), 4.42 - 4.29 (m, 1 H), 4.21 - 3.91 (m, 5 H), 3.77 (d, *J* = 10.4 Hz, 1 H), 3.58 - 3.44 (m, 3 H), 3.35 (s, 3 H), 2.57 - 2.43 (m, 1 H), 2.40 - 2.10 (m, 5 H), 2.09 - 2.00 (m, 3 H), 1.96 (d, *J* = 15.9 Hz, 1 H), 1.87 - 1.72 (m, 4 H), 1.72 - 1.54 (m, 3 H), 1.51 - 1.22 (m, 11 H), 1.19 - 1.07 (m, 5 H), 1.04 (dd, *J* = 7.0, 11.9 Hz, 7 H), 0.98 - 0.79 (m, 15 H) ^{13}C NMR (101MHz, CDCl3) 177.4, 160.6. 130.4, 121.3, 116.7, 108.2, 97.0, 85.8, 83.4, 82.4, 81.5, 78.0, 77.2, 74.4, 72.9, 69.0, 68.3, 60.3, 60.2, 57.4, 44.2, 42.5, 38.9, 37.4, 37.1, 35.7, 35.3, 35.1, 32.5, 32.2, 31.7, 30.7, 27.9, 27.5, 26.0, 25.7, 23.4, 22.6, 17.3, 16.3, 15.6, 14.2, 13.2, 13.1, 13.0, 10.8; (+) HRESIMS *m/z* 800.5075[M+H$^+$].

Analog 2(N-2): ^{1}H NMR (400MHz,CHLOROFORM-d) 7.1(d, J= 8.1 2H) 6.5 (d, J= 8.1 2H), 4.42 - 4.29 (m, 1 H), 4.20 - 3.91 (m, 5 H), 3.82 (d, *J* = 10.4 Hz, 1 H), 3.58 - 3.44 (m, 3 H), 3.35 (s, 3 H), 2.57 - 2.43 (m, 1 H), 2.40 - 2.10 (m, 5 H), 2.09 - 2.00 (m, 3 H), 1.96 (d, *J* = 15.9 Hz, 1 H), 1.87 - 1.72 (m, 4 H), 1.72 - 1.54 (m, 3 H), 1.51 - 1.22 (m, 11 H), 1.19 - 1.07 (m, 5 H), 1.04 (dd, *J* = 7.0, 11.9 Hz, 7 H), 0.98 - 0.79 (m, 15 H) ^{13}C NMR (101MHz, CDCl3) 177.4, 160.6. 130.4, 121.3, 116.7, 108.2, 97.0, 85.8, 83.4, 82.4, 81.6, 78.6, 77.6, 74.4, 72.6, 69.0, 68.7, 60.3, 60.2, 57.4, 44.2, 42.5, 38.59, 37.4, 37.5, 35.6, 35.8, 35.6, 32.6, 32.8, 31.6, 30.6, 27.7, 27.7, 26.0, 25.7, 23.4, 22.6, 17.3, 16.4, 15.4, 14.7, 13.6, 13.5, 13.3, 10.4; HRESIMS *m/z* 830.5180 [M+H$^+$].

Analog 3(N3):

^{1}H NMR (400MHz,CHLOROFORM-d) 7.33(m 2 H) 7.2 (m, 3 H), 4.41 - 4.23 (m, 1 H), 4.21 - 3.95 (m, 5 H), 3.84 (d, *J* = 10.4 Hz, 1 H), 3.54 - 3.43 (m, 3 H), 3.36 (s, 3 H), 2.54 - 2.43 (m, 1 H), 2.43 - 2.11 (m, 5 H), 2.06 - 2.01 (m, 3 H), 1.96 (d, *J* = 15.9 Hz, 1 H), 1.87 - 1.72 (m, 4 H), 1.72 - 1.54 (m, 3 H), 1.51 - 1.22 (m, 11 H), 1.19 - 1.07 (m, 5 H), 1.04 (dd, *J* = 7.0, 11.9 Hz, 7 H), 0.98 - 0.79 (m, 15 H) ^{13}C NMR (101MHz, CDCl3) 174.4, 158.5 (d, *J* = 164 Hz), 160.6. 130.3 (d, *J* = 10.3 Hz), 121.4, 116.5 (d, *J* = 19.2 Hz), 108.5, 97.0, 85.7, 83.6, 82.7, 81.4, 78.8, 77.5, 74.6, 72.4, 69.0, 68.4, 61.3, 61.4, 57.4, 44.4, 42.5, 38.4, 37.4, 37.1, 35.7, 35.6, 35.3, 32.2, 32.1, 31.1, 30.7, 27.9, 27.5, 26.0, 25.3, 23.3, 22.5, 17.5, 16.4, 15.4, 14.4, 14.2, 13.6, 13.4, 10.3; HRESIMS *m/z* 817.515 [M+H$^+$].

Analog 4(N-4): ^{1}H NMR (400MHz, CHLOROFORM-d) 7.33(m, 2H) 70 (m, 1H), 6.88 (dd, 8.1, 2.2 Hz 2H), 4.42 - 4.29 (m, 1 H), 4.24 - 3.95 (m, 5 H), 3.75 (d, *J* = 10.4 Hz, 1 H), 3.55 - 3.44 (m, 3 H), 3.35 (s, 3 H), 2.54 - 2.44 (m, 1 H), 2.40 - 2.14 (m, 5 H), 2.1 - 2.00 (m, 3 H), 1.96 (d, *J* = 15.9 Hz, 1 H), 1.86 - 1.74 (m, 4 H), 1.72 - 1.55 (m, 3 H), 1.51 - 1.22 (m, 11 H), 1.19 - 1.06 (m, 5 H), 1.05 (dd, *J* = 7.0, 11.9 Hz, 7 H), 0.98 - 0.79 (m, 15 H); ^{13}C NMR (101MHz, CDCl3) 175.4, 160.6. 130.4, 121.3, 116.7, 108.2, 97.0, 85.8, 83.4, 82.4, 81.5, 78.0, 77.2, 74.4, 72.9, 69.0, 68.3, 60.3, 60.2, 57.8, 44.8, 42.5, 38.9, 37.8, 37.2, 35.7, 35.7, 35.0, 32.9, 32.2, 31.4, 30.4, 27.8, 27.7, 26.0, 25.8, 23.7, 22.7, 17.7, 16.7, 15.7, 14.2, 13.9, 13.7, 13.3, 10.5; HRESIMS *m/z* 799.5234 [M+H$^+$].

Analog 5(N-5): ^{1}H NMR (400MHz,CHLOROFORM-d) 7.35(m 2 H) 7.5 (m, 1 H), 4.42 - 4.25 (m, 1 H), 4.24 - 3.96 (m, 5 H), 3.82 (d, *J* = 10.4 Hz, 1 H), 3.52 - 3.43 (m, 3 H), 3.36 (s, 3 H), 2.53 - 2.43 (m, 1 H), 2.43 - 2.17 (m, 5 H), 2.076 - 2.03 (m, 3 H), 1.98 (d, *J* = 15.9 Hz, 1 H), 1.87 - 1.72 (m, 4 H), 1.74 - 1.53 (m, 3 H), 1.51 - 1.21 (m, 11 H), 1.19 - 1.07 (m, 5 H), 1.04 (dd, *J* = 7.0, 11.9 Hz, 7 H), 0.98 - 0.79 (m, 15 H) ^{13}C NMR (101MHz, CDCl3) 174.4, 161.5 (d, *J* = 165.3 Hz), 160.6. 131.2 (d, *J* = 8.9 Hz), 121.4, 116.5 (d, *J* = 19.2 Hz), 97.1, 85.5, 83.6, 82.3, 81.2, 78.5, 77.6, 74.4, 72.4, 69.1, 68.2, 61.2, 61.5, 57.7, 44.7, 42.8, 38.4, 37.7, 37.1, 35.5, 35.7, 35.6, 32.1, 32.1, 31.5, 30.7, 27.9, 27.5, 26.0, 25.3, 23.3, 22.5, 17.5, 16.4, 15.4, 14.4, 14.2, 13.6, 13.4, 10.3; HRESIMS *m/z* 817.5154 [M+H$^+$].

S_ figure 9. HRMS date confirms the mass of nigricin derivative

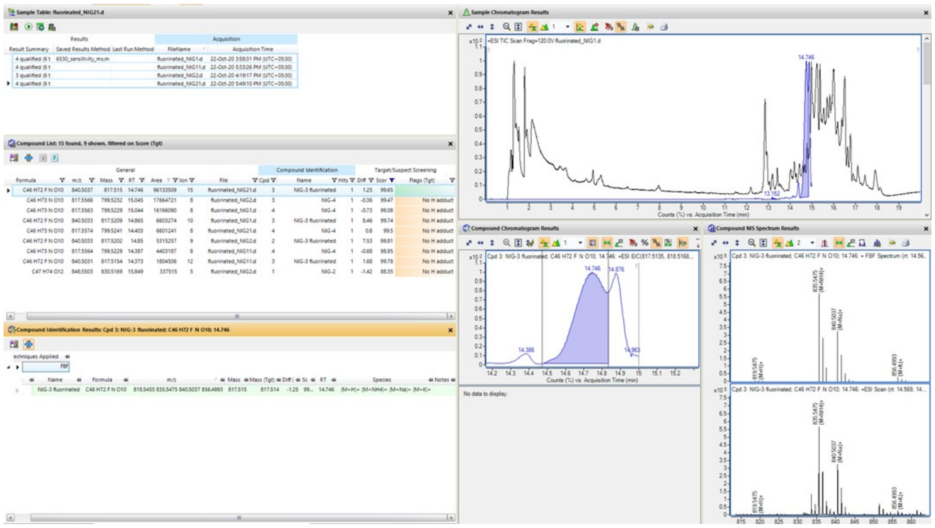

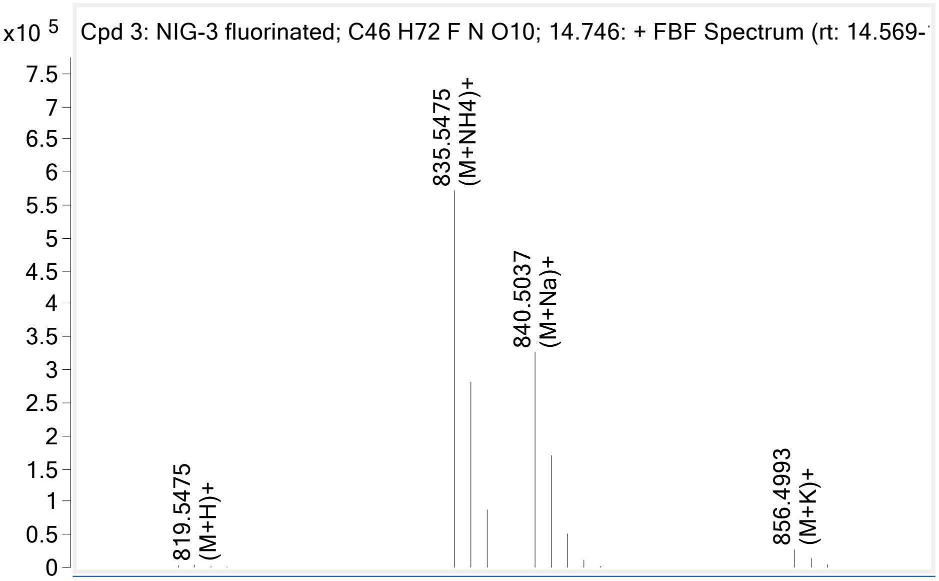

Species	Mono m/z
M (fluorinated nigricine) C46H72FNO10	817.514
(M+H)+	818.5213
(M+NH4)+	835.5479
(M+Na)+	840.5032
(M+K)+	856.4772

Bioactive potentials of Nigericin

Antibacterial activity of bioactive compound produced by *Streptomyces* sp. DASNCL29

S_ figure 10: Representative antibacterial activity against test organism *M.luteus, S.aureus and B.subtilis* and minimum inhibitory concentration of Nigericin. All experiments were performed in triplicate and all concentrations are in nM.

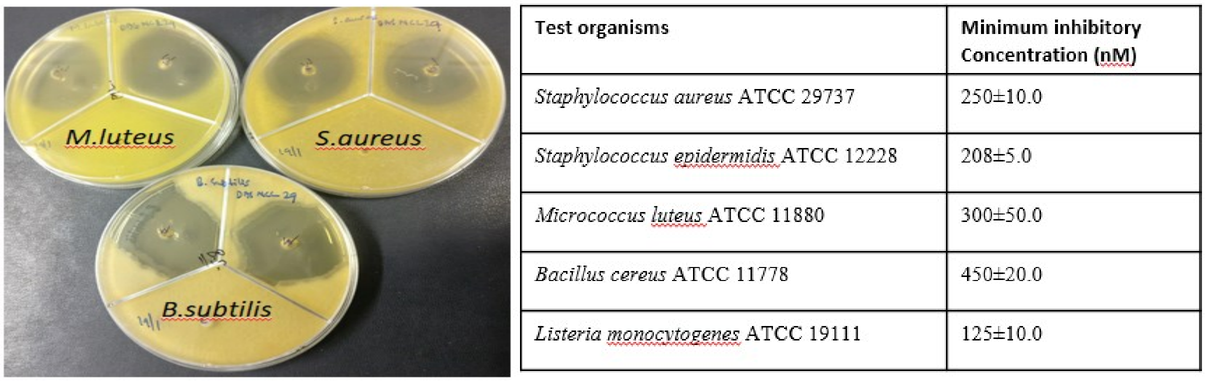

Test organisms	Minimum inhibitory Concentration (nM)
Staphylococcus aureus ATCC 29737	250±10.0
Staphylococcus epidermidis ATCC 12228	208±5.0
Micrococcus luteus ATCC 11880	300±50.0
Bacillus cereus ATCC 11778	450±20.0
Listeria monocytogenes ATCC 19111	125±10.0

S_Figure 10: Antibacterial activity of nigericin against *Staphylococcus aureus* (ATCC 29737). Nigericin produced by *Streptomyces* sp. DASNCL-29, Standard Nigericin (Sigma Aldrich, i.e. *S. hygroscopicus*) is used for comparison.

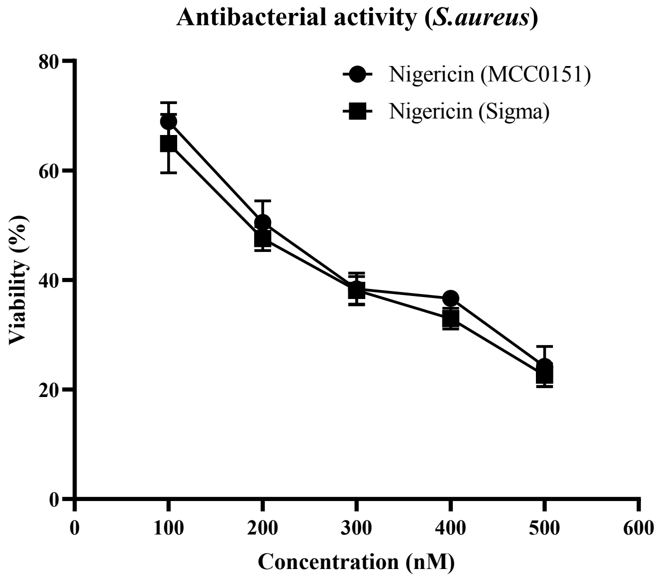

S_ figure 11: Transmission Electron Micrograph of test organism upon treatment with Nigericin and its fluorinated analogues. A and E are the control of *S. aureus* and *E. coli* respectively. B-D, is the morphology of treated cells of *S. aureus* with Nigericin, analogue-3 and analogue-5 respectively. F-H, is the morphology of treated cells of *E. coli* with Nigericin, analogue-3 and analogue-5 respectively

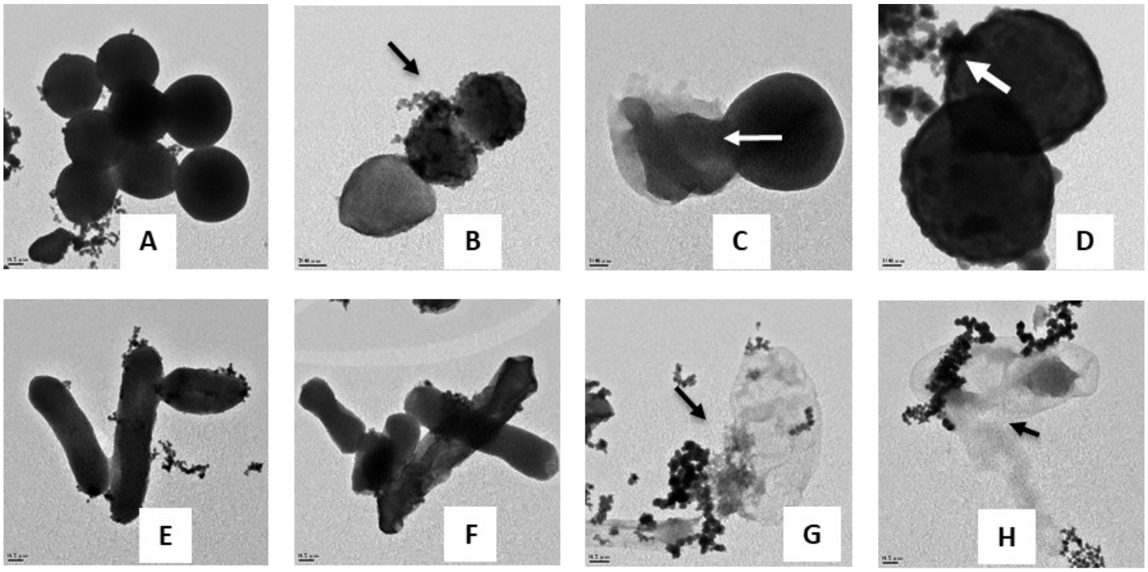

Antimalarial activity of bioactive compound produced by *Streptomyces* sp. DASNCL29

The O^{+ve} human blood, collected from anonymous donors and used for *P.falciparum* culture was purchased from the Poona Serological Trust Blood Bank, Pune, India. The experimental procedures for obtaining and using human blood were as per approved institutional guidelines.

Culturing *Plasmodium falciparum* blood-stage parasites: Asexual blood-stage P. falciparum (3D7 strain; obtained from MR4) parasites were cultured as previously reported (Subramanian *et al.,* 2018). Briefly, the parasites were grown using freshly obtained washed O^{+ve} human RBCs at 2.5% haematocrit in RPMI-HEPES medium at pH 7.4. The medium was supplemented with hypoxanthine (10mg/L), NaHCO3 (25mM), gentamicin (50mg/L), and AlbuMAX II (Thermo Fisher Scientific; 0.5% wt/vol).Mixed-stage parasite culture was synchronized using 5% sorbitol (Sigma-Aldrich) for the enrichment of ring-stage parasites (Radfar *et al*., 2009).

Parasite cultures were incubated with the inhibitors for 60 h under optimal growth conditions, following which the cells were lysed with 0.01% Triton X and stained with SYBR Green I nucleic acid stain (Thermo Fisher Scientific) to estimate parasite growth and inhibition. Fluorescence readings were obtained with a GloMax plate reader (Promega) after 15 min incubation in dark and raw fluorescence readings were processed. The % growth inhibition values were estimated from comparisons between test and control samples.

S_Table 7: Malaria parasite growth inhibition by Nigericin (Sigma) and 7B5 (Nigericin from Streptomyces sp DASNCL-29): Results from *in vitro* antimalarial activity testing of 7B5 and Nigericin are tabulated. The number of replicates n=3 for all samples.

Compound	Average % growth inhibition at 10 µM (± standard deviation)	*Antimalarial* EC_{50} (nM)
7B5 (Nigericin from DASNCL-29)	99.98±0.0	2.4±0.0
Nigericin (Sigma)	98.75±0.39	21.92±0.0

References

1 Yoon SH, Ha SM, Kwon S, Lim J, Kim Y, Seo H, et al. Introducing EzBioCloud: A taxonomically united database of 16S rRNA gene sequences and whole-genome assemblies. *Int J Syst Evol Microbiol*. 2017; **67**(5):1613–7.

2 Liu H, Du Z, Wang J, Yang R. Universal sample preparation method for characterization of bacteria by matrix-assisted laser desorption ionization-time of flight mass spectrometry. *Appl Environ Microbiol*. 2007; **73**(6):1899–907.

3 Freiwald A, Sauer S. Phylogenetic classification and identification of bacteria by mass spectrometry. *Nat Protoc.* 2009; **4**(5):732–42.

4 Matsuda N, Matsuda M, Notake S, Yokokawa H, Kawamura Y, Hiramatsu K, et al. Evaluation of a simple protein extraction method for species identification of clinically

relevant staphylococci by matrix-assisted laser desorption ionization - Time of flight mass spectrometry. *J Clin Microbiol*. 2012; **50**(12):3862–6.

5 Deng W, Nickle DC, Learn GH, Maust B, Mullins JI. ViroBLAST: A stand-alone BLAST web server for flexible queries of multiple databases and user's datasets. *Bioinformatics*. 2007; **23**(17):2334–6.

6 Blin K, Shaw S, Steinke K, Villebro R, Ziemert N, Lee SY, et al. AntiSMASH 5.0: Updates to the secondary metabolite genome mining pipeline. *Nucleic Acids Res*. 2019; **47**(W1):W81–7.
